# Quantifying wildfire impacts on atmospheric pollutants using fire exposure metrics and machine–deep learning approaches

**DOI:** 10.1038/s41598-026-51766-7

**Published:** 2026-05-09

**Authors:** Khushal Das, Sergio Flesca, Claudia Roberta Calidonna

**Affiliations:** 1 DIMES, University of Calabria, Rende CS, 87036 Italy; 2 CNR-ISAC Institute of Climate and Atmospheric Sciences, Lamezia Terme, Italy

**Keywords:** Wildfires, Atmospheric emissions, Fire exposure, Correlation analysis, Machine learning, Deep learning, Air quality forecasting, Climate sciences, Environmental sciences, Natural hazards

## Abstract

Wildfires are increasingly recognised as major contributors to atmospheric pollution, yet their spatial–temporal influence on regional air quality remains insufficiently understood. This study quantifies the impact of wildfire activity on atmospheric gas concentrations by integrating exposure modelling, correlation analysis, and advanced machine learning techniques. Two datasets were employed: (i) continuous atmospheric observations of CO, CO$_2$, CH$_4$, and BC alongside meteorological parameters; and (ii) a wildfire dataset containing fire locations, burned area, and duration. A novel Fire Exposure Index (FEI) was developed to quantify the dynamic likelihood that wildfire plumes influence the observatory, incorporating fire distance, burned area, and wind characteristics. Correlation analyses across distance bands and daily lags (up to six days) revealed clear distance and wind-dependent relationships, with short-term lag effects primarily within one to two days. Baseline machine learning models (Gradient Boosting, Random Forest, Decision Tree) achieved moderate accuracy, while recurrent neural networks (LSTM, GRU, BiLSTM) captured stronger temporal dependencies, particularly for CO$_2$ and CO. A stacked ensemble architecture was subsequently implemented, combining LightGBM, LSTM, GRU, and BiLSTM with a LightGBM meta-learner. The hybrid framework achieved substantial performance improvements within the study domain ($\textrm{R}^2$ values ranging from 0.959 to 0.9897 across CO, CH$_4$, and CO$_2$), demonstrating strong predictive capability under the specific meteorological and geographic conditions examined. The proposed approach demonstrates that integrating exposure metrics with hybrid ensemble learning provides a promising and interpretable strategy for predicting wildfire-induced atmospheric variability and supporting air quality management in fire-prone regions.

## Introduction

Wildfires are among the major contributors to global air quality deterioration, significantly increasing atmospheric pollution and posing serious risks to human health and environmental sustainability^1^. Naturally, fires have been taking place almost in all biomes and have been important in the formation of ecosystems and landscapes. As humans spread across the world, fire became a component of their agricultural activities, land management, and cattle keeping. In the modern world, people are the primary source of fire outbreaks, and it is estimated that over 90% of all fires in the world are either deliberately or accidentally set by humans^2,3^. Climate change has increased the frequency, duration, and severity of wildfires in many regions due to rising temperatures, altered rainfall patterns, and prolonged droughts. The mixture of gases and particulate matter (carbon monoxide (CO), carbon dioxide (CO$_2$), methane (CH$_4$), black carbon (BC), volatile organic compounds, and fine particles) released by the wildfires can travel great distances, interact with the meteorological conditions, and affect the local and regional air quality^4^. Wildfires lead to significant economic and environmental losses, threaten human lives and contribute around 17% of the global CO$_2$ emissions^5^. With unique climatic conditions, vegetation type, and the accelerated prevalence of wildfires caused by climate change, the Mediterranean area has become an essential region in comprehending the links of wildfire emissions to climate change and the environment^6^. Italy is located in the core of the Mediterranean basin and is prone to the occurrence of wildfires that affect the atmospheric structure, wildfire emissions are difficult to characterise because they arise from the complex interaction of meteorological conditions, fuel properties, combustion processes, and atmospheric transport mechanisms. Pollutants such as CO, CO$_2$, CH$_4$, BC, and volatile organic compounds (VOCs) can travel long distances and influence air quality far from the original fire location^7^. Traditional methods of understanding these relationships have developed from basic source characterisation studies to advanced integrated observation-modelling systems using state-of-the-art analytical techniques^8^. The combination with correlation analysis, machine learning, and deep learning methods has changed our capabilities for the prediction and modelling of the effects that wildfires have on atmospheric emissions with incredible insights regarding emission patterns and their effects on the environment^9^. However, despite progress, few studies have systematically compared traditional ensemble methods with temporal deep learning architectures in the context of wildfire-induced gas emissions^10^. Moreover, the integration of static ensemble learners with recurrent temporal networks remains underexplored, especially for forecasting multi-gas atmospheric variability in complex Mediterranean environments. Our study advances this frontier by developing a hybrid stacked ensemble that combines Gradient Boosting and LightGBM with Long Short-Term Memory (LSTM), Gated Recurrent Unit (GRU), and Bidirectional LSTM (BiLSTM) models to jointly exploit both static and sequential dependencies in atmospheric signals.

The technological development of satellite methods, the ground-based surveillance system, and the processing power have made researchers develop a more advanced system of modelling the wildfire emissions. The joint success of correlation analysis, where the statistical relationship can be established between fire characteristics and emission patterns and machine learning methods, deep learning models, which are capable of identifying complex nonlinear relationships, represent a paradigm shift in environmental modelling^11^. It is especially applicable to Italy, where an irregular topography and complicated meteorological conditions demand more analytical delicacy.

Climate change is intensifying wildfire activity in the Mediterranean, with Italy expected to experience longer fire seasons, larger burned areas, and greater emissions^12^. While most studies focus on particulate matter (PM2.5), fewer examine gaseous pollutants and their temporal behavior. Understanding pollutant transport and lag effects is essential for accurate air quality forecasting, yet traditional exposure metrics often overlook key meteorological factors such as wind speed and direction that strongly influence pollutant dispersion^13,14^.

This study combines both correlation analysis and machine learning and deep learning techniques to simulate the effects of the wildfire activity on atmospheric emissions at an observatory distant enough not to be in the fire, including a proposed stacked ensemble architecture that fuses LightGBM with temporal deep learners (LSTM, GRU, BiLSTM) and employs a LightGBM meta-learner to combine base predictions. The interest in various pollutants and the development of a new Fire Exposure Index that unites the distance to fire, the size of the burned area, and the wind parameters should help to present a more in-depth picture of wildfire emissions and their impact on air quality, as the study will consider. The main contributions of this paper are:

1. **Fire Exposure Index (FEI):** Development and systematic evaluation of a multi-formulation Fire Exposure framework that integrates alternative spatial decay structures, directional weighting mechanisms, temporal persistence terms, and normalization strategies to quantify wildfire influence on atmospheric observations.
2. **Temporal Lag Analysis:** A systematic analysis of the pollutant responses with respect to the fire occurrence for different time lag (up to 6 days) and distance bands to determine prevalent transport and delay effects.
3. **Multi-Pollutant Assessment:** Detailed analysis of several gaseous species such as CO, CO$_2$, CH$_4$ and BC to obtain an integrated knowledge of wildfire emission and transport patterns.
4. **Machine and Deep Learning Prediction:** Development and comparison of different predictive algorithms (Gradient Boosting, Random Forest, Decision Tree, LSTM, GRU, and BiLSTM) using the Fire Exposure Index (FEI) and meteorological covariates as predictors in order to evaluate their individual prediction ability.
5. **Proposed Stacked Ensemble Architecture:** Design and evaluation of a hybrid stacking framework that integrates LightGBM, LSTM, GRU, and BiLSTM as base learners under a LightGBM meta-learner. This architecture effectively captures both nonlinear static relationships and temporal dependencies, achieving markedly higher accuracy ($R^2> 0.98$ for CO, CH$_4$, and CO$_2$; $R^2 = 0.93$ for BC) compared with individual models.

## Literature review

### Wildfire emissions and atmospheric pollutants

Studies have established that wildfires emit a complex blend of gases and particulate materials, and thus they are significant pollutants in the atmosphere and cause atmospheric quality and climate change. CO, CO$_2$, CH$_4$, BC, volatile organic compounds (VOCs), and particulate matter (PM 2.5) are some of the principal pollutants^15,16^. These emissions also cause the development of the ground-level ozone and secondary organic aerosols, which affect the regional and global atmospheric chemistry.

Recent studies have highlighted the minimised role of wildfires in global CH$_4$ emissions. Zhao et al.^17^ utilised satellite inversion techniques to demonstrate that global CH$_4$ emissions from fires have historically been underestimated by as much as 27%, primarily due to unresolved small fires and low spatial resolution in emission inventories. This highlights the necessity for more accurate emission inventories that account for the full spectrum of wildfire activity.

Further underlining this point, a study by Filonchyk et al.^18^ examined air quality implications of the wildfires in Canada from May 11–14, 2024, using ground-based data and satellite monitoring. Their findings suggest that traditional emission inventories may underestimate the contributions of minor and transient wildfire events to regional air pollution levels.

Additionally, the 2024–2025 fire season^19^ was the second-highest fire year on record for burned area and carbon emissions in the North American boreal forests. Despite a relatively low area burned, global fire carbon emissions reached 2.2 Pg C, 9% above average and the sixth highest on record, driven by intense, high-emission fires in South America and Canada.

These studies collectively highlight the need for refined emission inventories and modelling approaches that more accurately capture the full spectrum of wildfire activity, including minor and transient events, to better assess their impacts on atmospheric composition and air quality.

### Transport and temporal dynamics of wildfire emissions

Several meteorological phenomena affect the transport of wildfire emissions such as the speed and direction of the wind, which have an influence on the dispersion and deposition of the pollutants with time^20^. Cristofanelli et al.^21^ used information available in Integrated Carbon Observation System (ICOS) locations across Europe to compute the contributions of wildfire events to CO$_2$ variability in the background in several years and observed that fire perturbations were present in background concentrations over several years. Likewise, Zheng et al. generated unprecedented levels of CO2 emissions during boreal fires in the year 2021, which is the highest in living memory, and also represents the increasing percentage of Northern fires in global carbon cycles^22^.

It is, therefore, necessary to understand the time dynamics of the transport of the pollutants if one is to optimally develop a forecast of the air quality. The research work of Liao et al.^23^ modeled the transport of CO2 of a large wildfire in Canada. They found that meteorological drivers such as wind speed and vertical motion play an integral role in the explanation of delays in concentrations observed by them^23^. These results demonstrate how important the consideration of temporal lags and meteorological considerations are to the modeling of wildfire emission effects.

### Exposure metrics and modelling approaches

Traditional exposure metrics typically rely on variables such as fire proximity and burned area to estimate the potential impact of wildfire emissions. However, these metrics often fail to capture important meteorological factors, such as wind direction and wind speed, which strongly influence pollutant dispersion. In reaction to this, Kinase et al.^3^ introduced what they call a Fire Exposure Index which is a mixture of the closeness of fire, fire burnt area and wind parameters as a more suitable measurement of the likelihood for wildfire emissions to affect the measurements of a far station.

Machine learning (ML) and deep learning (DL) approaches have been increasingly applied to predicting the outcomes of air quality in terms of wildfire emissions. A review by Considine et al.^16^ compared high resolution models of PM2.5 (meteorological) derived data and that these models missed extreme cases of smoke, particularly during peak wildfire events. It implies that ML models need to be tweaked to the conditions of exposure to wildfires and improved with new predictors in order to mitigate spikes of pollutants. Recent studies have increasingly adopted ensemble machine learning approaches for wildfire prediction across diverse geographic regions. For example, Ahajjam et al.^24^ proposed a wildfire prediction framework for Alaska that integrates spatio-temporal clustering of satellite-derived fire observations with ensemble machine learning models to predict wildfire occurrence, burned area, and fire duration. Their approach combines remote sensing data, geospatial variables, and feature-selection optimization to improve predictive accuracy across multiple forecasting horizons. The results demonstrate that ensemble learning architectures can substantially enhance wildfire prediction performance compared with individual machine learning models. Similar ensemble-based frameworks have also been applied in other regions to model wildfire susceptibility and fire behaviour using heterogeneous environmental predictors. These developments highlight the growing role of ensemble and hybrid modelling strategies in wildfire research. However, most existing studies focus primarily on wildfire occurrence or burned-area prediction, whereas comparatively fewer investigations integrate physically informed exposure metrics with atmospheric pollutant forecasting, particularly within Mediterranean environments. The present study contributes to this area by coupling wildfire exposure modelling with hybrid machine–deep learning architectures for atmospheric gas prediction.

Additionally, various studies have proposed models that can be used to measure wildfire emission and transportation. As an example, National Fire-Danger Rating System (NFDRS) provides the equations for calculating the potential of the fire by meteorological parameters, like direction and speed of wind^25^. Also, there is the Atmospheric Dispersion Index (ADI) that quantifies the wind transport vector which is the average speed and direction between the earth and mixing height to estimate the movement of smoke^26^.

Despite these advancements, there are some limitations to the current models. As an illustration, the NFDRS is based on the assumptions of steady-state conditions and not a spatial variability in the types of fuels that can influence fire behaviour^25^. On the same note, the ADI uses observations of surface and upper air, which might not reflect fine-scale variations in wind patterns^26^. Equally, the ADI depends on surface and upper air, which are not capable of reflecting fine-scale changes in the wind patterns. Such constraints necessitate the development of more sophisticated models that capture meticulously meteorological information that consider spatial heterogeneity in fuel and terrain properties. Further, fire emissions are linked to respiratory and cardiovascular diseases, as well as premature mortality. A study by Xu et al.^27^ estimated that the 2023 wildfire season in Canada led to over 87,000 premature deaths worldwide due to air pollution, with health effects observed even in regions far from the fire locations.

Conventional measures of exposure and methods of modelling offer good insights on the wildfire emissions and their effects on air quality. More effective predictions and management strategies should include meteorological variables and consider the model limitations. This illustrates the health impacts of wildfire smoke worldwide and effective measures should be taken to monitor and manage it.

## Datasets

This work incorporates five years of data, between January 2019 and December 2023, combining the records of wildfire occurrences, weather observations and the release of atmospheric emissions in Calabria, southern Italy. These datasets jointly offer a strong basis to examine wildfire dynamics, weather variability and pollutant behaviour across five years of time, which can be used to create higher-order machine learning and deep learning models to measure forecasting wildfire behaviour and environmental evaluation when analysing environmental support^28^.

**Fig. 1:**
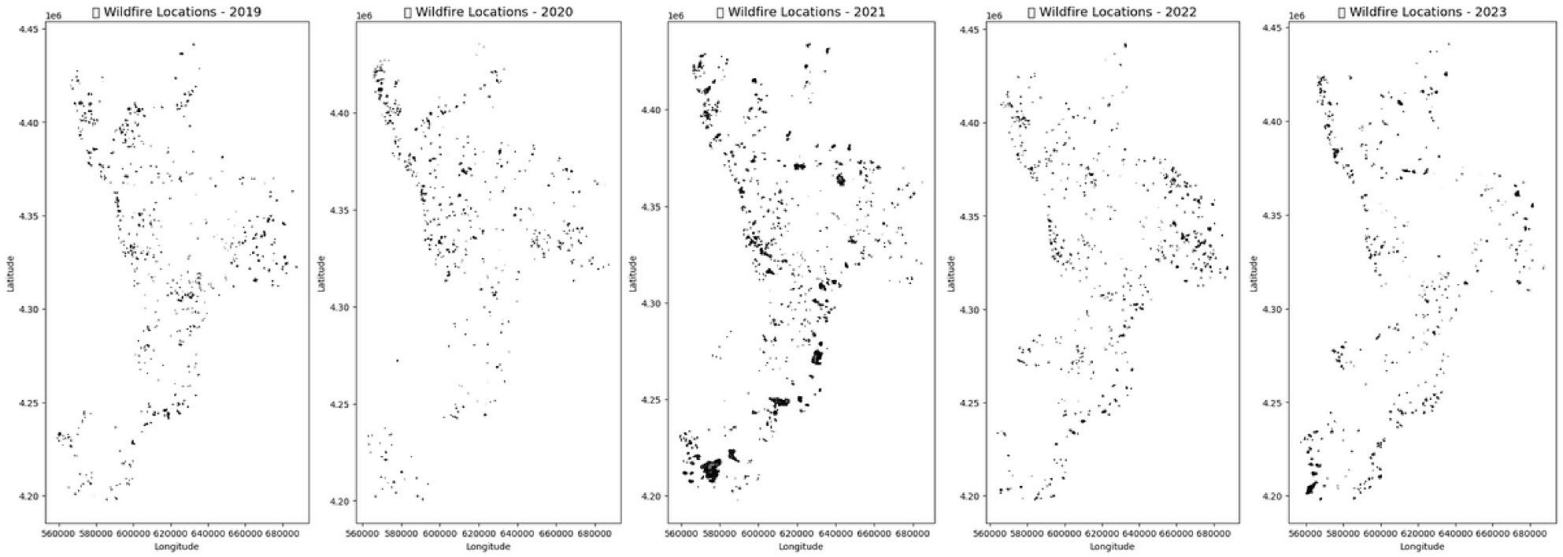
Graphical representation of 2019-2023 wildfire events in the Calabria Region of Italy.

### Wildfires events record

The wildfire events dataset was used as the main ground truth in the process of identifying and characterizing fires in Calabria. The records contain accurate spatial and time information, such as the time when the fire broke out and its time of completion, coordinates of latitude and longitude, and area burnt, which enables the exact monitoring of the progress and spread of individual fires. There was a significant change in the wildfire activity in 2019-2023: 1,271 fires in 2019, 1,108 in 2020, the maximum in 2021 (1,863 fires) in the conditions of extended drought and high temperatures, 1,395 in 2022 and 1,181 in 2023^29^. Data cleaning was performed by excluding minor or poorly documented fire events. The cleaned dataset consists of 6,818 confirmed wildfire incidences, which offer a powerful basis of data labelling, integration, and model training. Figure 1 presents the spatial distribution of all wildfire events recorded between 2019 and 2023. The x axis represents the longitude and the y axis represents the latitude which shows the spatial heterogeneity and clustering of the fire occurrences over the region.

### Meteorological dataset

**Fig. 2:**
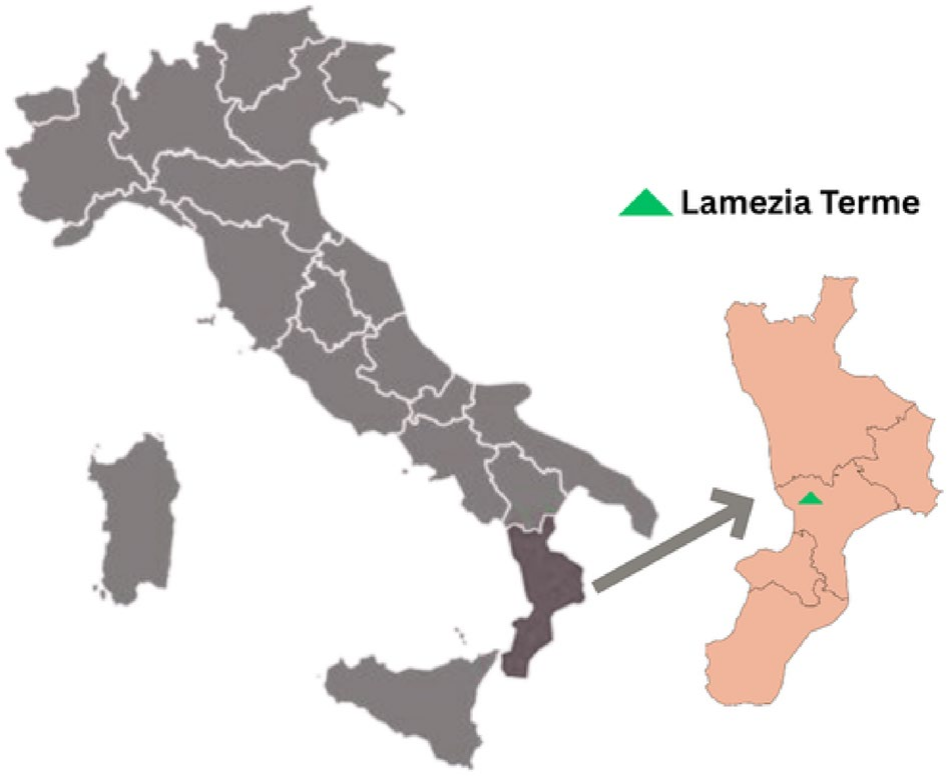
Observatory location, Lamezia Terme, Italy.

The wildfire occurrence data is supplemented by the meteorological dataset, which is a record of the Lamezia Terme Observatory in Calabria region as presented in Fig. 2. Originally captured at 1-minute intervals, this dataset was subsequently aggregated to a 1-hour temporal resolution to highlight broader atmospheric patterns associated with larger and more intense wildfire events, while minimising short-term fluctuations unrelated to fire dynamics. The resulting dataset comprises approximately 43,800 hourly records spanning the five years.

All the records contain important meteorological variables such as wind speed and direction, temperature, pressure, and relative humidity, and these parameters describe mesoscale conditions affecting wildfire ignition, spread and plume dispersal. This resolution allows analysis of atmospheric variations before and after wildfire events. In the case of supervised learning meteorological data were marked by wildfire records in the three-step process. To begin with, all the timestamps were rated using binary labels (1 = active fire and 0 = no fire). Second, the geodesic distances between the observatory and fire events were computed and summed up into ranges (0-20km, 20-40km, 40-60km, 60-80km, 80-100km, 100-120km, 120-140km, and >140km). Third, to categorize the fires, the azimuthal angles were calculated to come up with eight cardinal directions (N, NE, E, SE, S, SW, W, NW). This multi-level labelling transformed raw meteorological data into spatially distributed inputs that are passed on to machine and deep learning models to forecast occurrence of wildfires, direction, and proximity.

### Atmospheric emissions dataset

The Atmospheric Emissions Dataset consists of continuous, ground-based in situ measurements collected at the Lamezia Terme Observatory 2 between 2019 and 2023, originally recorded at 1-minute resolution and subsequently aggregated to hourly averages for alignment with meteorological and wildfire data.

The dataset includes calibrated concentrations of BC, CO, CO$_2$, and CH$_4$, along with standard deviations and calibration parameters to ensure accuracy. These indicators provide critical insight into combustion intensity, plume composition, and fire-related atmospheric transformations, complementing meteorological variables with direct chemical evidence of wildfire influence.

After synchronization and integration with meteorological and wildfire occurrence data, a unified matrix of 2,601,320 hourly observations was created, labelled by fire occurrence (binary), fire distance range, and fire direction. Of these, 1,522,400 entries corresponded to non-fire periods and 1,078,920 to active fire events. Although the dataset provides extensive temporal coverage and high-resolution measurements, it is derived from a single observatory and therefore primarily captures local atmospheric conditions, which may limit the representation of broader spatial variability in wildfire–atmosphere interactions.

This spatially labeled, high-resolution, multi-year dataset offers a promising empirical foundation for training and validating machine and deep learning models to infer wildfire presence, predict fire direction, and estimate exposure effects. Its integration of meteorological and chemical parameters enables a comprehensive understanding of fire–atmosphere interactions, supporting improved wildfire forecasting and risk mitigation in southern Italy.

## Methods

### Correlation analysis

We investigated the association between wildfire activity and atmospheric composition at the *Lamezia Terme Observatory* using a suite of correlation designs built on the hourly analysis grid. Let $X_{v,t}$ denote the emission measured at hour *t* for variable $v\in \{\textrm{BC},\textrm{CO},\mathrm {CO_2},\mathrm {CH_4}\}$. For each wildfire event *i* active at hour *t* we know its burned area $A_i$ (ha), duration $D_i$ (days), great-circle distance $d_i$ (km) from the observatory, and bearing $\phi _i$ (deg). The hourly wind direction at the observatory is $\psi _t$ (deg), with $\Delta \theta _i(t)$ the minimal circular difference between $\phi _i$ and $\psi _t$ (deg; converted to radians inside trigonometric functions). Distance bins are defined as,

$$\mathcal {B}=\{[0,20),[20,40),[40,60),[60,80),[80,100),[100,120),[120,140),[140,\infty )\}\;\text {km}.$$

The eight directional sectors correspond to $\{\textrm{N},\textrm{NE},\textrm{E},\textrm{SE},\textrm{S},\textrm{SW},\textrm{W},\textrm{NW}\}$, with central azimuths $\{0^\circ ,45^\circ ,\dots ,315^\circ \}$ and sector width $45^\circ$.

**Binary indicators by direction and distance.** For each hour *t*, direction $k\in \{1,\dots ,8\}$, and distance bin $b\in \mathcal {B}$, we form a Boolean indicator that equals one if *any* fire is active in that sector and range:

1 $$\begin{aligned} I_{k,b}(t)= {\left\{ \begin{array}{ll} 1, & \exists \, i \text { active at } t \text { with } \phi _i\in \text {sector }k \text { and } d_i\in b,\\ 0, & \text {otherwise.} \end{array}\right. } \end{aligned}$$

The sector-only indicator $I_k(t)$ used below is the logical “or” across bins: $I_k(t)=\textbf{1}\{\sum _{b} I_{k,b}(t)\ge 1\}$.

**Correlation coefficient and lags.** All correlations are computed as Pearson’s *r* on the hourly grid; for binary covariates (e.g., $I_{k,b}$), this coincides with the point-biserial correlation. For a lag of *L* *days* we correlate $Y_t$ with $Z_{t+24L}$, where 24 converts days to hours. Formally,

2 $$\begin{aligned} r_{Y,Z}(L)= \frac{\textrm{cov}\!\left( Y_t,\; Z_{t+24L}\right) }{\sqrt{\textrm{var}(Y_t)\,\textrm{var}(Z_{t+24L})}}. \end{aligned}$$

We evaluate $L\in \{0,1,\dots ,6\}$ to probe effects up to six days after an event hour. Before computing (2), each series is aligned to the standard set of valid timestamps; missing hours are removed pairwise. For figures that compare across variables, we also report correlations using *z*-scored emissions $\tilde{X}_{v,t}=(X_{v,t}-\mu _v)/\sigma _v$ (mean $\mu _v$ and standard deviation $\sigma _v$ computed on the analysis window).

#### Fire_1–Fire_8 correlations by distance (binary design)

To quantify whether the presence of fires within specific distance categories is associated with contemporaneous or delayed changes in emissions, we compute

3 $$\begin{aligned} \begin{aligned} r^{\text {dist}}_{k,v}(L) = \textrm{corr}\!\left( I_{k}(t),\, X_{v,t+24L}\right) , \qquad k=1,\dots ,8,\;\; v,\;\; L=0{:}6 . \end{aligned} \end{aligned}$$

Here, $I_k(t)$ is a binary indicator representing wildfire occurrence within the *k*-th distance category relative to the observatory. This formulation produces correlation profiles describing how atmospheric emission responses vary as a function of wildfire proximity over subsequent days.

#### Fire_1–Fire_8 correlations by day-lag

To analyse temporal lag effects for each wildfire distance category, we compute

4 $$\begin{aligned} \begin{aligned} r_{k,v}(L) = \textrm{corr}\!\left( I_{k}(t),\, X_{v,t+24L}\right) , \qquad k=1,\dots ,8,\;\; v,\;\; L=0{:}6 . \end{aligned} \end{aligned}$$

The resulting lag profiles describe how pollutant concentrations respond to wildfire activity occurring at different proximity ranges over subsequent days.

#### Area-burned influence on observed emissions

To assess intensity effects, we form an hourly area signal either by direction or overall. The directional version sums areas of all active fires within sector *k* (across bins) at hour *t*:

5 $$\begin{aligned} \begin{aligned} S_k(t)=\sum _{i \in \mathcal {A}_t \cap \text {sector }k} A_i, \qquad S(t)=\sum _{k=1}^{8} S_k(t). \end{aligned} \end{aligned}$$

We then compute correlations at daily lags using either $S_k(t)$ or *S*(*t*):

6 $$\begin{aligned} \begin{aligned} r^{\text {area}}_{k,v}(L)=\textrm{corr}\!\left( S_k(t),\, X_{v,t+24L}\right) , \qquad r^{\text {area}}_{v}(L)=\textrm{corr}\!\left( S(t),\, X_{v,t+24L}\right) , \quad L=0{:}6. \end{aligned} \end{aligned}$$

Because $S_k(t)$ is right-skewed, we additionally report sensitivity using $\log \!\big (1+S_k(t)\big )$.

#### Daily-lag correlations for each directional sector

For completeness, and to match the “Fire_1 to Fire_8 at daily lags” design, we summarize (??) as sector-specific lag profiles $\{r^{\text {dir}}_{k,v}(L)\}_{L=0}^{6}$ for every $k\in \{1,\dots ,8\}$ and *v*; these are visualized as line plots over *L* to reveal peak lag and decay.

#### Correlations with the five exposure formulas

Let $\textrm{Exposure}_m(t)$, $m=1,\dots ,5$, be the hour-*t* exposure metrics defined in Equations 8–12. We examine both concurrent and delayed associations with emissions via

7 $$\begin{aligned} \begin{aligned} r^{\text {exp}}_{m,v}(L) = \textrm{corr}\!\left( \textrm{Exposure}_m(t),\, X_{v,t+24L}\right) , \qquad m=1,\dots ,5,\;\; v,\;\; L=0{:}6. \end{aligned} \end{aligned}$$

Since exposures are continuous and may vary in scale across *m*, we also report correlations using standardised exposures $\tilde{E}_{m}(t)=(\textrm{Exposure}_m(t)-\mu _m)/\sigma _m$ to facilitate cross-formula comparison.

All correlations use pairwise-complete hours after aligning the covariate and response series under lag *L*. Reported *p*-values are based on the usual *t*-statistic for Pearson’s *r* with $n-2$ degrees of freedom; because hourly series exhibit serial dependence, we complement these with confidence intervals from a stationary block bootstrap using 24-hour blocks, which preserves diurnal structure. Multiple comparisons across sectors, bins, lags, variables, and formulas are addressed by controlling the false discovery rate with the Benjamini_Hochberg procedure at $\alpha =0.05$. Results are presented as heatmaps for (??) and (7), lag-profile plots for, and bar or line summaries for (6); all figures use the same hourly base and the same valid-hour mask to ensure comparability.

### Exposure calculation

To capture different possible mechanisms through which wildfire emissions influence atmospheric observations, five alternative exposure formulations were developed and evaluated. Each formulation represents a different physical assumption about plume transport and persistence. Exposure1 emphasizes strong distance attenuation and directional wind alignment for nearby fires. Exposure2 introduces event duration and a slower spatial decay to represent longer-lasting fire influence. Exposure3 incorporates temporal ageing to reduce the contribution of older plumes. Exposure4 models directional alignment using cosine weighting while jointly considering fire size and duration. Finally, Exposure5 provides a normalized composite index that balances fire size, duration, wind speed, and distance through scale-invariant weighting. Together, these formulations allow systematic comparison of alternative exposure hypotheses and enable identification of the most predictive representation of wildfire influence on atmospheric measurements.

We quantify hour-*t* exposure at the *Lamezia Terme Observatory* using five alternative metrics that combine wildfire intensity, proximity, wind magnitude and alignment, and (where relevant) event persistence and age. For each wildfire event *i*, let $A_i$ denote the total burned area (ha) and $D_i$ its duration (days) from first detection to containment; let $d_i$ be the great-circle distance (km) from the observatory to the fire centroid, and $\phi _i$ the corresponding bearing (degrees clockwise from north). Hourly meteorology at the observatory provides wind speed $u_t$ (m s$^{-1}$) and wind direction $\psi _t$ (degrees clockwise from north). The minimal circular angular difference between a fire’s bearing and the wind direction is

$$\Delta \theta _i(t) \;=\; \min \!\big (|\phi _i-\psi _t|,\; 360^\circ -|\phi _i-\psi _t|\big ),$$

which is converted to *radians* whenever it appears inside $\exp (\cdot )$ or $\cos (\cdot )$. The active set $\mathcal {A}_t$ comprises all fires whose active window overlaps hour *t* and, to capture lingering smoke, those within 72 hours after containment. We also define $\textrm{Age}_i(t)=t-t_{\text {start},i}\ge 0$ (days) as time since ignition. Distances are computed geodesically (haversine) together with initial azimuths for $\phi _i$; distances smaller than a numerical threshold $\varepsilon =1$ km are replaced by $\varepsilon$ to avoid singularities in formulas with $1/d_i$ or $1/d_i^2$. Hourly wind direction is obtained via vector (circular) averaging of minute-level directions; hourly speed is the arithmetic mean of minute-level speeds. Unless stated otherwise we adopt the constants $\sigma =15^\circ$ (converted to radians where used), $\theta _{\max }=45^\circ$, $\lambda =0.3$, and $d_{50}=50$ km. All metrics are computed on an hourly time base aligned to the wildfire timeline and the post-containment tail.

#### Inverse distance with Gaussian directional weighting

8 $$\begin{aligned} \textrm{Exposure}_{1}(t) = \sum _{i\in \mathcal {A}_t} \left( \frac{A_i\,u_t}{d_i^{2}} \right) \exp \!\left( -\frac{\Delta \theta _i(t)^{2}}{2\,\sigma ^{2}}\right) . \end{aligned}$$

This metric emphasises downwind transport from nearby high-intensity fires. The $1/d_i^2$ term reflects rapid dispersion-like decay with distance, while multiplying by $u_t$ increases exposure under more substantial advection. The Gaussian factor imposes smooth directional selectivity around the downwind axis, with $\sigma$ controlling how narrowly alignment is rewarded.

#### Linear directional weight, log duration, and 1/d decay

9 $$\begin{aligned} \begin{aligned} \textrm{Exposure}_{2}(t) = \sum _{i\in \mathcal {A}_t} \left( \frac{A_i\,u_t\,\ln \!\big (1+D_i\big )}{d_i} \right) \left( 1-\frac{\Delta \theta _i(t)}{\theta _{\max }}\right) _{+}, \qquad (x)_{+}=\max (x,0). \end{aligned} \end{aligned}$$

Here, the spatial decay is slower ($1/d_i$), which is suitable when the influence extends farther. Event persistence is represented through the term $\ln (1+D_i)$, rewarding longer events with diminishing returns. The directional weighting decreases linearly with angle and is truncated to zero at $\theta _{\max }$, producing an interpretable cone of influence.

#### Age-decay, power-law duration, Gaussian directional weight

10 $$\begin{aligned} \begin{aligned} \textrm{Exposure}_{3}(t) = \sum _{i\in \mathcal {A}_t} \left( \frac{A_i\,u_t\,D_i^{1/2}}{d_i} \right) \exp \!\left( -\frac{\Delta \theta _i(t)^{2}}{2\,\sigma ^{2}}\right) \exp \!\big (-\lambda \,\textrm{Age}_i(t)\big ). \end{aligned} \end{aligned}$$

This variant retains a broader spatial footprint ($1/d_i$) but discounts older plumes with an exponential age term. The sublinear $D_i^{1/2}$ rewards persistence without allowing very long events to dominate. Directional sensitivity remains Gaussian as in (8).

#### Cosine directional alignment and area–duration over distance squared

11 $$\begin{aligned} \textrm{Exposure}_{4}(t) = \sum _{i\in \mathcal {A}_t} \left( s \frac{A_i\,D_i\,u_t}{d_i^{2}} \right) \cos \!\big (\Delta \theta _i(t)\big ). \end{aligned}$$

The cosine term provides a signed alignment measure: perfect alignment ($0^\circ$) yields 1, cross-wind ($90^\circ$) gives 0, and counter-wind contributions are negative. This can be informative when counter-aligned flow should explicitly penalise exposure. If non-negativity is required, a clipped alternative $\max \{0,\cos (\Delta \theta _i(t))\}$ can be reported as a sensitivity analysis.

#### Normalised composite index with half-distance decay

12 $$\begin{aligned} \textrm{Exposure}_{5}(t) = \sum _{i\in \mathcal {A}_t} \left( \frac{A_i}{A_{\max }} \cdot \frac{D_i}{D_{\max }} \cdot \frac{u_t}{u_{\max }} \cdot \frac{\cos \!\big (\Delta \theta _i(t)\big )}{1+\big (d_i/d_{50}\big )^{2}} \right) , \end{aligned}$$

**Fig. 3:**
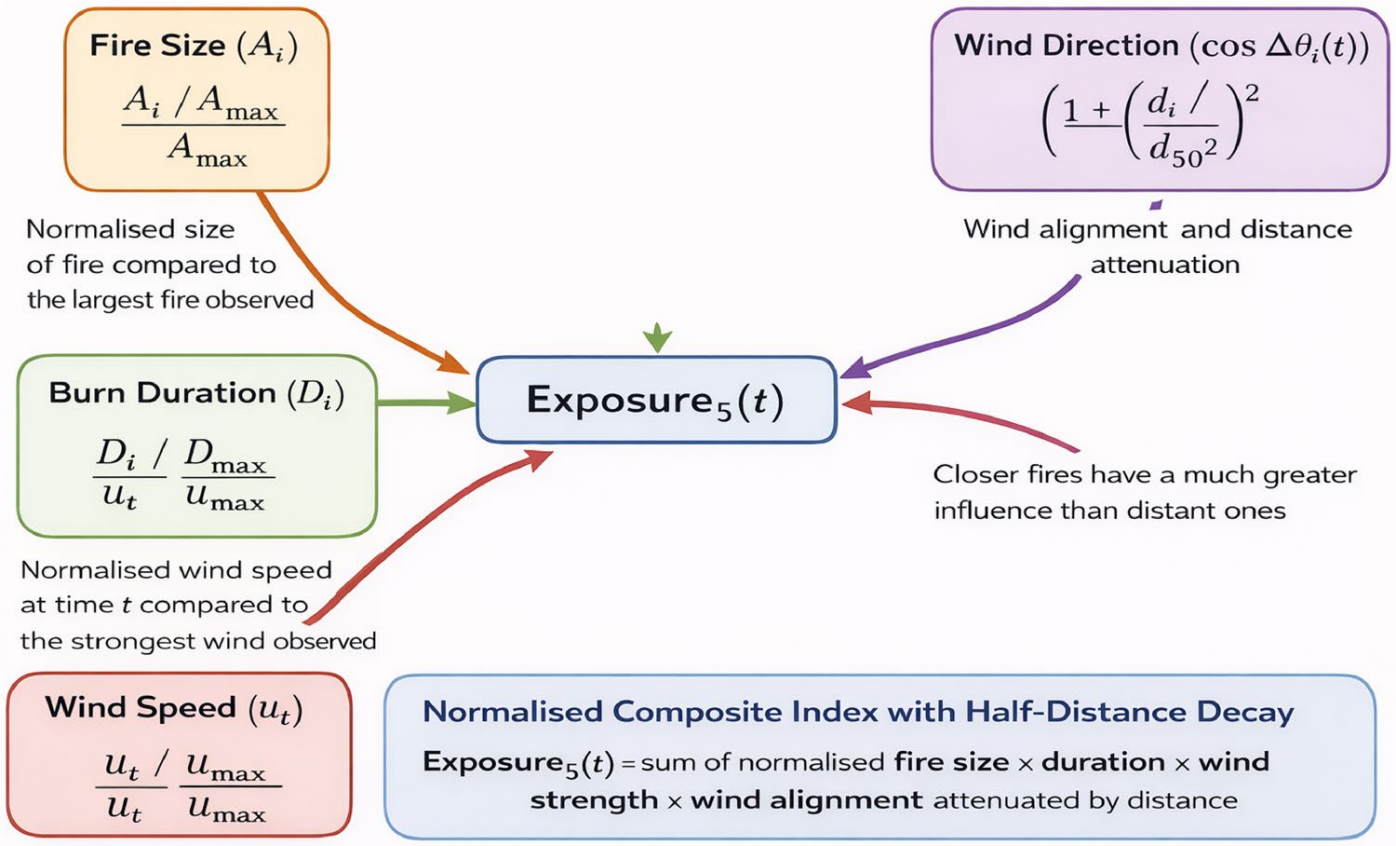
Schematic representation of the Normalised Composite Fire Exposure Index with half-distance decay, illustrating the combined contribution of fire size, burn duration, wind speed, wind direction alignment, and distance attenuation to the overall exposure at time t.

Where $A_{\max }$, $D_{\max }$ and $u_{\max }$ are the maxima computed on the training folds (and then fixed for validation/testing to avoid information leakage), and $d_{50}$ is the characteristic half-distance (the distance at which the denominator doubles and the weight halves). The result is a dimensionless scale-invariant index that is directly comparable across hours, seasons, and years. The Fig. 3 shows conceptual diagram of the Normalised Composite Fire Exposure Index with half-distance decay, illustrating how fire intensity, duration, wind conditions, and spatial attenuation jointly determine exposure at time t.

#### Implementation details

For each hour *t*, we construct $\mathcal {A}_t$ by intersecting event timelines with *t* and appending a 72-hour post-containment tail to reflect residual smoke. The distances $d_i$ and bearings $\phi _i$ are geodesically calculated from the observatory to each fire centroid; minimal distances are replaced by $\varepsilon =1$ km to stabilise the divisions. This threshold was introduced as a numerical stabilization step when computing exposure formulations that include inverse-distance terms (e.g., $1/d_i$ and $1/d_i^{2}$). When distances approach zero, these terms can become unrealistically large and lead to numerical instability during exposure aggregation. In practice, wildfire centroids were never located exactly at the observatory site; however, very small computed distances may arise due to spatial resolution and centroid approximation of fire polygons. To avoid singularities while maintaining realistic spatial weighting, the small constant threshold ($\varepsilon = 1$ km) was applied. This stabilization approach is consistent with standard practices in spatial interaction modelling and inverse-distance weighting methods, where small-distance thresholds are introduced to prevent singular behaviour while preserving physically meaningful decay patterns.

Hourly wind direction is obtained by vector-averaging minute-level directions (unit-vector mean with atan2); hourly wind speed is the arithmetic mean of minute-level speeds. Angular differences $\Delta \theta _i(t)$ are computed as minimal circular differences in degrees and converted to radians inside trigonometric functions. This approach ensures that wind variables used in the exposure formulation reflect aggregated atmospheric conditions relevant to plume transport rather than short-term fluctuations. All five exposure metrics are then evaluated on the same hourly grid used for the labelled meteorological and emissions datasets, ensuring temporal alignment with wildfire activity and the post-containment window. Normalisation constants for (12) are estimated on the training portion of the data within each cross-validation split and reused on held-out folds to prevent look-ahead bias. Sensitivity analyses, such as varying $\sigma$, $\theta _{\max }$, $\lambda$, or $d_{50}$, or clipping the cosine in (11), can be reported, but unless otherwise noted, the default values above are used throughout.

### Machine and deep learning models

To establish baseline predictive performance, a series of traditional ensemble learning models and deep sequential architectures were implemented and optimized. The inclusion of both model families enables the comparison of non-temporal regression algorithms against time-aware recurrent neural networks, providing a comprehensive understanding of model behavior under dynamic atmospheric conditions.

#### Tree-based ensemble models

The traditional machine learning part contained three popular ensemble regressors namely Gradient Boosting Regressor (GBR), Decision Tree (DT), and Random Forest (RF). These models were chosen because they are proven to be able to deal with nonlinear relationships and complicated feature interactions. To determine the best configuration of hyperparameters according to the cross-validation performance, each algorithm was fine-tuned by systematic grid searching.

The Decision Tree model was the baseline non-parametric learner and could model hierarchical feature splits and it overfits if used individually. To overcome this, Random Forest algorithm was introduced, which makes use of bootstrap aggregation (bagging) of multiple randomized trees to decrease variance and to increase generalization of the model. The Gradient Boosting Regressor was further able to improve the performance by successively creating weak learners to fix the residual of previous models that improved the convergence towards the overall target function. Together, these models incorporate complementary strengths: interpretability (based on tree structures), stability (based on ensemble averaging), and incremental error reduction (based on boosting).

The input features for these models included non-temporal atmospheric and exposure indicators including wind speed, wind direction and weighted exposure score. The models were trained in order to predict hourly gas concentration values for CO, CH$_{4}$, CO$_{2}$ and BC. The predictability of each model was tested using Root Mean Square Error (RMSE) and the coefficient of determination ($R^2$), at least to be able to compare different target gases.

#### Deep sequential models

In addition to ensemble regressors, three deep learning models–Long Short-Term Memory (LSTM), Bidirectional LSTM (BiLSTM), and Gated Recurrent Unit (GRU)–were implemented to capture temporal dependencies in gas concentration time series. Unlike static models, these recurrent neural networks (RNNs) learn sequential patterns, enabling prediction of pollutant levels from preceding meteorological and exposure states using a one-step-ahead forecasting approach.

LSTM employs memory cells with input, forget, and output gates to manage long-term dependencies, while BiLSTM extends this by processing data in both forward and backward directions to better capture complex temporal relationships. GRU, a simplified variant, combines gating mechanisms for faster convergence and reduced computational cost.

All models were optimized using the Adam optimizer (learning rate = $10^{-3}$) with MSE loss, dropout regularization, and early stopping for generalization. Performance was evaluated on a hold-out test set for direct comparison with ensemble models. Whereas ensemble regressors effectively captured nonlinear feature interactions, RNNs excelled at modeling temporal autocorrelation, motivating their integration in the hybrid stacked ensemble framework described in Section "Proposed stacked ensemble architecture".

### Proposed stacked ensemble architecture

Taking advantage of an individual model’s strengths, we have designed a hybrid stacking ensemble architecture which combines both tree-based and deep sequential learning model. The proposed model combines four base learners - Light Gradient Boosting Machine (LightGBM), LSTM, GRU, and Bidirectional LSTM (BiLSTM) under the umbrella of meta-learning with LightGBM considered as the final meta-learner.

**Stage 1: Base Learners** Each base learner is trained separately over the same input set of features so as to predict the target gas concentrations. Compared to LSTM, GRU and BiLSTM, which all utilize sequential data, LightGBM utilizes leaf-wise gradient boosting for providing good generalization for tabular data. The BiLSTM in particular traverses sequences back and forth improving temporal context-sensitivity.

**Stage 2: Out-of-Fold Predictions** To avoid meta-learning bias, a TimeSeriesSplit cross-validation was used. Under typical scenarios, each base learner will calculate OOF predictions on unseen data partitions, that is, an independent estimate of the target variable. These OOF predictions are used as meta-predictions, which in a sense summarize the strengths of all the base models. A time-aware cross-validation framework (TimeSeriesSplit) was employed to preserve temporal ordering and prevent information leakage. In this approach, each validation fold strictly follows the training data in time, ensuring that predictions are generated on unseen future observations. However, we acknowledge that validation remains confined to a single observatory and regional context, and therefore does not fully capture cross-regional generalization.

**Stage 3: Meta-Learner Training** The meta-learner (LightGBM) is trained taking the OOF predictions as input features and the real target values as targets. This stage discovers the optimum nonlinear combination of base model outputs balancing out their complementary behaviours. The meta-learner was optimized by early stopping by validation loss.To prevent overfitting, the meta-learner was trained only on out-of-fold predictions generated from validation folds using a TimeSeriesSplit strategy, ensuring that base model predictions were always produced from models trained on temporally earlier data.

**Stage 4: Final Prediction** In the inference stage, the trained base learners make predictions for new samples which are then fed into the meta-learner, to make the stacked output. This approach is effective combining short-term sequence learning with usage of ensemble-based generalization allowing for improved predictive accuracy with less sensitivity to overfitting.

**Fig. 4:**
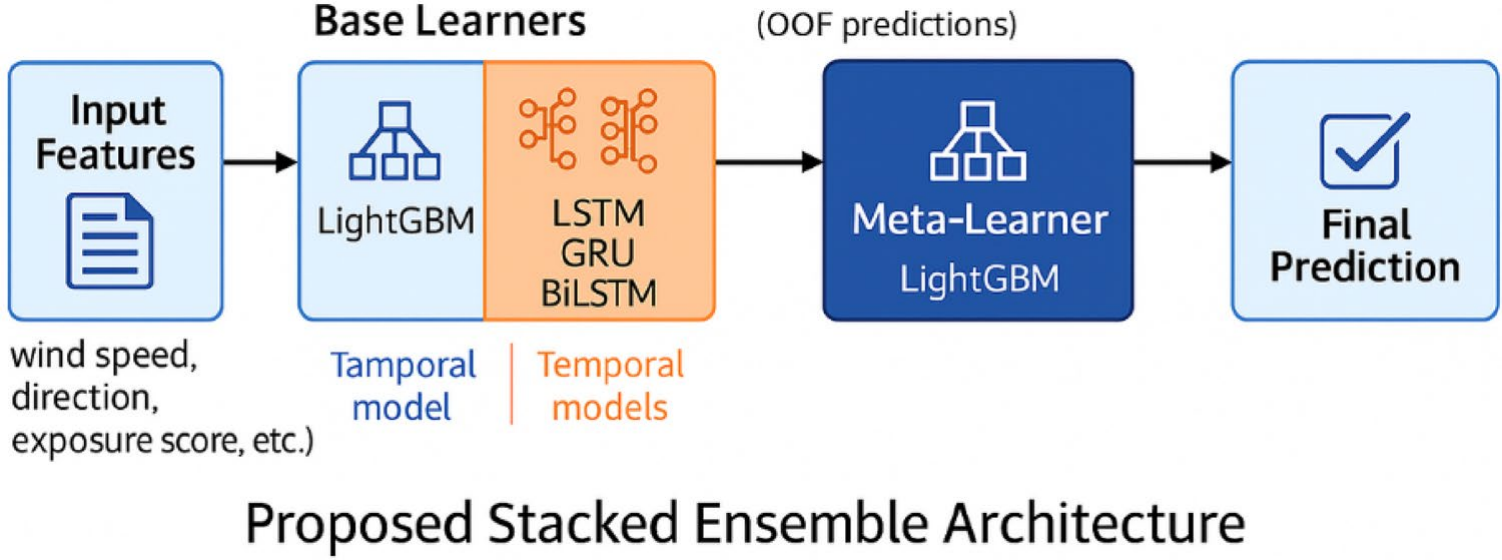
Proposed stacking ensemble pipeline integrating tree-based and deep sequential base learners.

The performance of all models, including the stacked ensemble, was evaluated using root mean square error (RMSE) and coefficient of determination ($R^2$). The stacked architecture achieved superior performance by capturing both nonlinear tabular interactions and temporal dependencies within the gas concentration time series (Fig. 4).

## Results

### Correlation findings

**Fig. 5:**
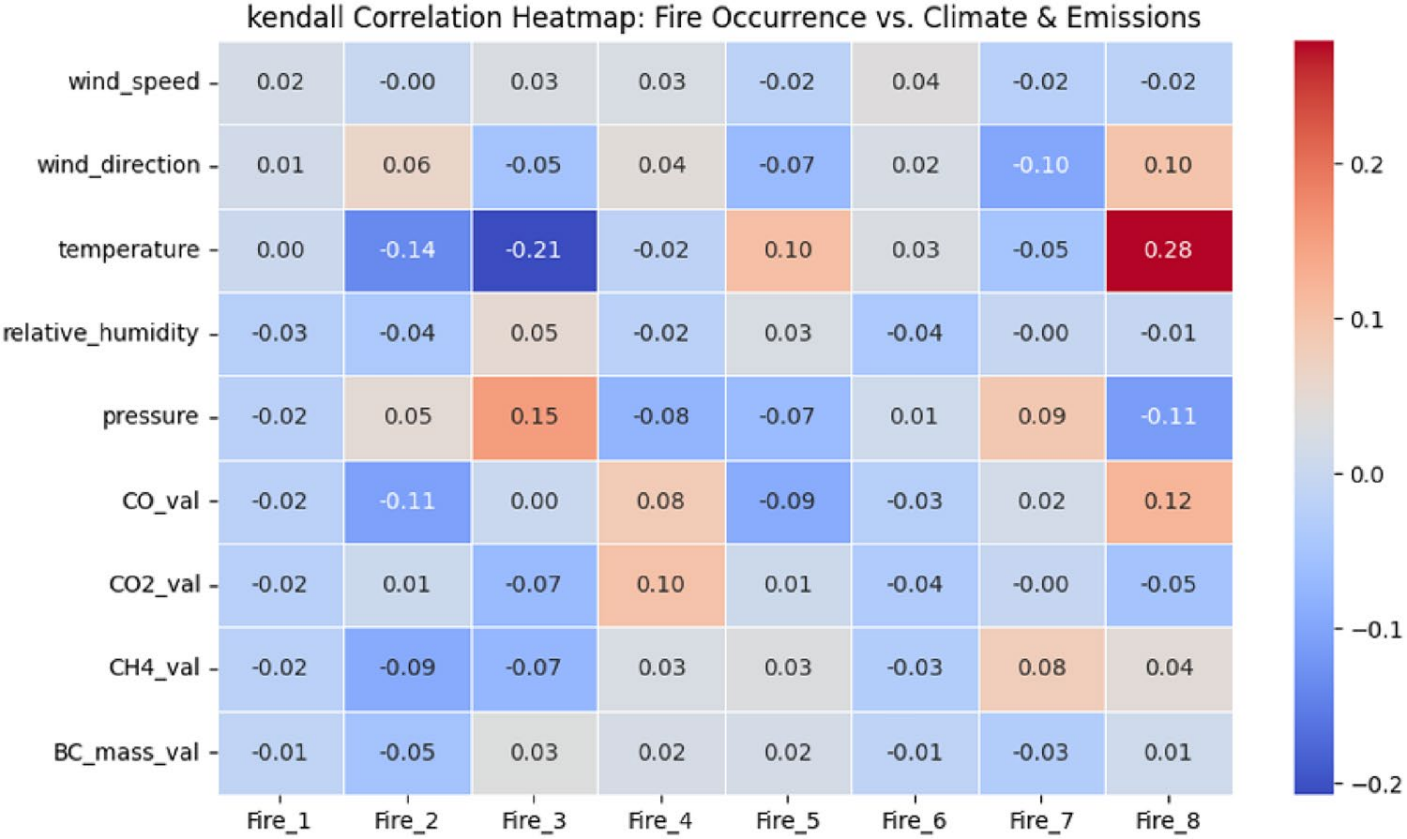
Correlation between wildfire occurrence (binary 1/0) and emission parameters based on distance intervals from the observatory.

The first stage evaluated direct relationships between wildfire occurrences and atmospheric emissions at the Lamezia Terme Observatory using a binary labelling scheme (1 = fire present, 0 = no fire). Correlations were computed separately by distance band (Fire_1: 0–20 km; Fire_2: 20–40 km; Fire_3: 40–60 km; etc.), producing the matrix shown in Fig. 5. Results were generally weak to moderate across distance categories, with the strongest positive values for nearby fires (0–20 km), indicating that proximal fires tend to influence CO, CO$_2$, CH$_4$, and BC but that the simple presence/absence signal is limited. The attenuation with distance is consistent with plume dilution and prevailing wind-driven dispersion.

These limited correlations likely reflect several shortcomings of the binary approach and observational constraints: the binary label ignores fire size, duration and intensity; there is uncertainty from spatial and temporal mismatches between fire events and the observatory’s sampling intervals; and local meteorological variability (changing wind patterns, atmospheric pressure fluctuations, and vertical mixing) can mask or redirect plumes. Together, these factors explain why a fixed-point, binary analysis yields only modest signals and motivate more nuanced exposure-based metrics and modelling.

**Fig. 6:**
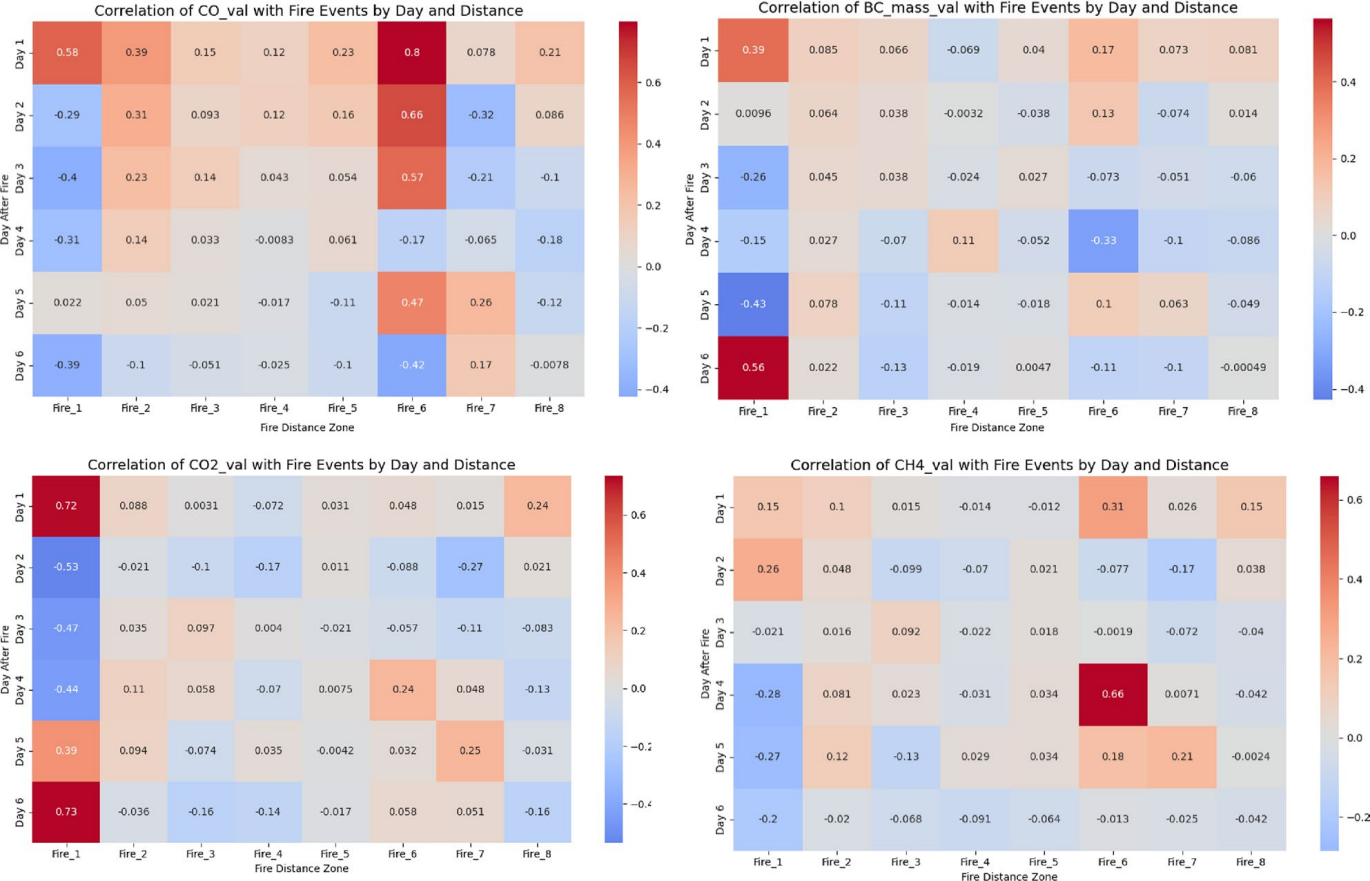
Day-based lagged correlations showing the effect of wildfire occurrence on emissions at the observatory from Day 1 to Day 6 after fire events.

To further refine the temporal interpretation, a second correlation analysis was performed by introducing day-based lags between fire events and emission responses. In this approach, correlations were computed for the same distance-based fire indicators (Fire_1 to Fire_8) but analysed over successive days following each fire event (Day 1, Day 2, Day 3, and so on). The results are presented in Fig. 6. This figure includes four correlation diagrams representing emissions at daily lags relative to fire occurrence.

**Fig. 7:**
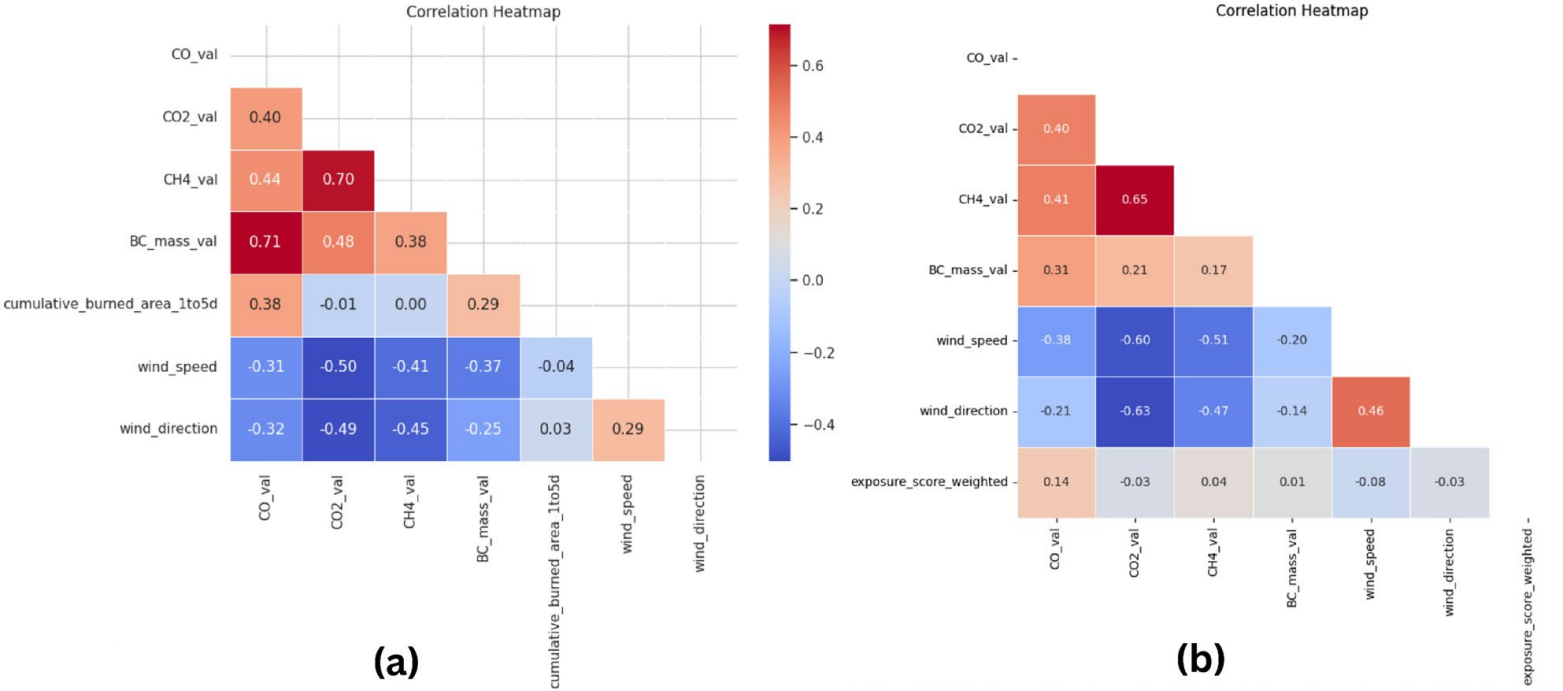
(**a**) Correlation of emission parameters with total burned area, showing variable relationships influenced by fire intensity and meteorological dispersion. (**b**) Correlation heatmap showing relationships between the Fire Exposure Score and emission variables at the Lamezia Terme Observatory. Positive values indicate higher pollutant levels when fires are nearby and wind-aligned.

The day-lagged analysis tested whether weak same-day correlations were due to delayed plume transport. Slight increases on Days 1–2, particularly for CO and BC, suggested short-term effects from nearby fires, while declining trends afterward reflected rapid dispersion under changing wind and mixing conditions. Incorporating burned area improved physical interpretation by accounting for fire intensity, though correlations remained inconsistent due to terrain, vegetation, timing mismatches, and meteorological variability. Overall, the results shown in Fig. 7-(a) showed that simple correlations cannot fully capture fire–emission dynamics, supporting the need for more advanced exposure-based and machine learning models.

Following the previous correlation analyses based on distance, temporal lag, and burned area, a more refined approach was developed to better represent the combined influence of fire intensity, proximity, and meteorological conditions on emissions measured at the Lamezia Terme Observatory. Recognising that wind plays a critical role in determining whether fire plumes reach the observation point, a new variable, referred to as the Fire Exposure Score, was introduced. This metric integrated four key parameters: total burned area ($A_i$), distance from the observatory ($d_i$), wind speed ($u_t$), and the angular difference between wind direction and fire direction ($\Delta \theta _i$). The exposure score was computed as follows:

13 $$\begin{aligned} \text {Exposure}_{\text {weighted}} = \left( \frac{A_i}{d_i^2} \right) \times \left( 1 - \frac{|\text {wind\_deg} - \text {fire\_deg}|}{45} \right) \end{aligned}$$

The daily cumulative exposure value was then obtained by summing the weighted exposure scores from all fires active during the same day:

14 $$\begin{aligned} \text {Daily Exposure Score} = \sum _{\text {fires active on day}} \text {Exposure}_{\text {weighted}} \end{aligned}$$

In this formulation, the first term $\left( \frac{A_i}{d_i^2} \right)$ emphasises the intensity of nearby fires by giving higher weight to large fires occurring closer to the observatory. The second term $\left( 1 - \frac{|\text {wind\_deg} - \text {fire\_deg}|}{45} \right)$ adjusts the contribution of each fire according to wind direction alignment. When the fire direction closely aligns with the prevailing wind direction (i.e., $\Delta \theta _i \approx 0^\circ$), the weighting term approaches 1, indicating maximum exposure as smoke plumes are directly transported toward the observatory. Conversely, when the angular difference exceeds $45^\circ$, the weighting term diminishes toward zero, reflecting minimal or no expected impact because the wind transports emissions away from the measurement site.

The refined Daily Fire Exposure Score integrates fire proximity, size, and wind conditions, offering a more realistic representation of plume transport. Correlation results showed moderate positive links between exposure and emissions, strongest for CO$_2$ (r = 0.40) and CH$_4$ (r = 0.41), indicating higher pollutant levels when fires were nearby and wind-aligned. Strong CO$_2$–CH$_2$ co-variation (r = 0.65) reflected similar combustion sources. Negative correlations with wind speed and misaligned wind direction confirmed that stronger or opposing winds dilute or divert plumes. Overall, incorporating meteorological and spatial factors improved model realism, revealing that short-range, wind-aligned fires most strongly affect local air quality, while atmospheric variability limits correlation strength. The correlation results between this newly defined *Daily Exposure Score* and the emission parameters are presented in Fig. 7-(b).

### Fire exposure performance

**Fig. 8:**
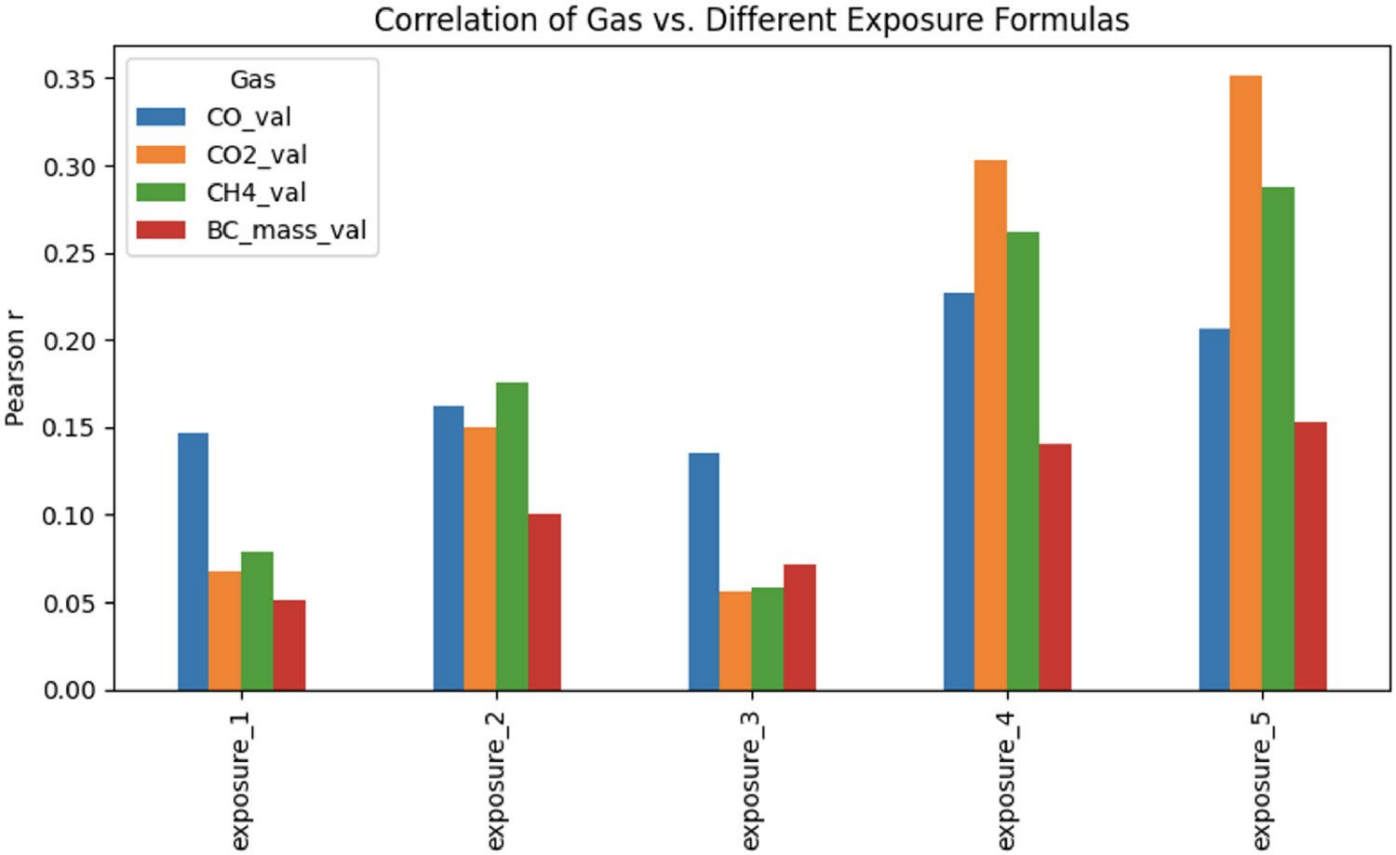
Correlation of gas concentration variables (CO, CO$_2$, CH$_4$, BC_mass) with five Fire Exposure formulations (*Exposure*$_1$–*Exposure*$_5$). The progressive enhancement in correlation indicates that composite exposure metrics incorporating area, duration, wind speed, and direction better explain observed variability in emission measurements.

Building upon the earlier wind- and distance-weighted exposure formulation, five alternative *Fire Exposure* metrics (*Exposure*$_1$ to *Exposure*$_5$) were evaluated to assess their ability to capture the atmospheric response to wildfire activity. Each exposure variant, as defined in the methodological section, represents a unique physical hypothesis regarding how plume transport and intensity contribute to observed gas concentrations at the Lamezia Terme Observatory. Figure 8 presents the Pearson correlation coefficients between each exposure formulation and the four principal gas variables: CO, CO$_2$, CH$_4$, and BC_mass.

**Exposure_1: Inverse Distance with Gaussian Directional Weighting.** The first formulation emphasises the influence of nearby fires under strong downwind alignment. As shown in Fig. 8, this metric yielded modest correlations across all gas variables, with slightly higher association for CO ($r \approx 0.15$). The limited magnitude suggests that while proximity and direction capture some immediate fire impacts, the exclusion of temporal persistence and ageing limits its explanatory power for prolonged emission influence.

**Exposure_2: Linear Directional Weight, Log Duration, and 1/** *d* **Decay.** Introducing event duration improved performance moderately, as reflected by increased correlations for CH$_4$ ($r \approx 0.18$) and CO$_2$ ($r \approx 0.17$). The inclusion of $\ln (1+D_i)$ allowed long-lasting fires to exert sustained influence without overweighting extreme durations. This adjustment better represents cumulative exposure but remains sensitive to distance variability, explaining the moderate correlation levels observed.

**Exposure_3: Age-Decay, Power-Law Duration, Gaussian Directional Weight.** The third formulation incorporated temporal ageing through an exponential decay factor, reducing the contribution of older plumes. However, this additional weighting caused a slight decrease in correlations across all gases, indicating that the exponential decay may have suppressed delayed transport effects that still influence air quality. Consequently, this version captured plume persistence less effectively than expected, particularly for CO and CH$_4$.

**Exposure_4: Cosine Directional Alignment and Area–Duration over Distance Squared.** The fourth formulation produced the highest correlation improvements, especially for CO$_2$ ($r \approx 0.30$) and CH$_4$ ($r \approx 0.26$). By integrating both fire size and duration through the $A_i D_i$ term, while maintaining inverse-square distance weighting, this metric more accurately modelled the combined spatial and temporal influence of large, sustained fires. The cosine directional alignment term effectively differentiated between plume-aligned and counter-wind events, improving the physical realism of exposure estimation.

**Exposure_5: Normalized Composite Index with Half-Distance Decay.** The final formulation achieved the strongest overall performance across all gases, particularly CO$_2$ ($r \approx 0.35$) and CH$_4$ ($r \approx 0.29$). Its normalisation over maximum area, duration, and wind speed produced a dimensionless, scale-invariant exposure index suitable for inter-annual comparison. The inclusion of a half-distance decay function $\big (1+(d_i/d_{50})^2\big )^{-1}$ provided smoother spatial weighting, capturing both near- and mid-range fire contributions. The consistent improvement in correlation across pollutants demonstrates that this formulation best represents the combined physical and meteorological dynamics influencing pollutant transport.

The progressive improvement of the model with the help of the temporal persistence, fire ageing, wind alignment, and normalization parameters prove the high interpretability and predictability of the fire exposure measures. The beneficial performance differences between one formulation and the next, although overall correlations were moderate because of atmospheric dispersion and meteorological variations, confirm the strength of the method. Exposure 5 was the most meaningful and coherent across all the gas species, proving to be the most informative and physically sensible variable of the effects of wildfires at the Lamezia Terme Observatory and the variable of interest to be utilized in further predictive research.

**Physical Consistency:** The behaviour of the proposed Fire Exposure Index (FEI) is consistent with known atmospheric transport mechanisms. First, stronger correlations are observed under wind-aligned conditions (Fig. 7-(b)), supporting the role of directional advection in plume transport. Second, the attenuation of correlations with increasing distance reflects expected dispersion effects. Third, the presence of short-term lag responses (1–2 days) is consistent with transport and mixing timescales reported in previous atmospheric studies.

### Machine and deep learning models outcomes

**Fig. 9:**
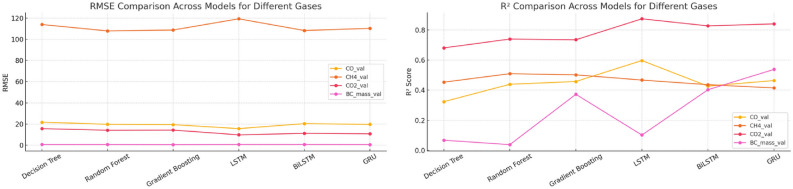
Comparison of $R^2$ (top) and RMSE (bottom) values across all machine and deep learning models for CO, CH$_4$, CO$_2$, and BC prediction.

To establish comparative baselines across the individual learning architectures, both traditional machine learning and deep recurrent models were evaluated on the prediction of four atmospheric gas concentrations (CO, CH$_4$, CO$_2$, and BC). Performance was assessed using the coefficient of determination ($R^2$) and root mean square error (RMSE). Table 1 and Fig. 9 summarize the key findings.

**Tree-based ensemble results** Among the traditional regressors, Random Forest and Gradient Boosting consistently outperformed the Decision Tree model across most target gases. For CO$_2$, the Random Forest achieved the highest performance ($R^2=0.74$, RMSE=14.18), followed closely by Gradient Boosting ($R^2=0.73$, RMSE=14.31). Both models demonstrated the ensemble advantage of variance reduction through bagging and error correction through boosting. In contrast, the single Decision Tree exhibited weaker generalization, with lower $R^2$ scores (0.32–0.68) and higher RMSE values across all gases.

**Deep learning results** The recurrent neural network architectures (LSTM, BiLSTM, and GRU) demonstrated stronger ability to model temporal dependencies within meteorological and exposure time series data. For CO$_2$, the LSTM achieved the highest overall predictive accuracy ($R^2=0.87$, RMSE=9.81), followed closely by GRU ($R^2=0.84$, RMSE=10.78). BiLSTM also performed competitively ($R^2=0.83$, RMSE=11.22). For CO and BC, the GRU yielded marginally better performance than LSTM and BiLSTM, indicating its suitability for shorter-term dependencies and lower computational overhead. CH$_4$ prediction remained more challenging due to higher variance and nonlinear temporal behavior, where all models exhibited relatively modest $R^2$ values (0.41–0.47).

Overall, the deep learning models outperformed tree-based ensembles in capturing the temporal dynamics of CO$_2$ and CO, while ensemble regressors maintained robustness for gases with higher short-term variability. The prolonged dominance of LSTM and GRU for long-horizon gas forecasting was the methodological starting point to throw in more colors to the stacked ensemble framework proposed in this paper, bridging models to have an improved generalization ability and robustness (Section 5.4).

**Table 1:** Performance summary ($R^2$ and RMSE) of baseline machine and deep learning models for all target gases.

Model	CO ($R^2$/RMSE)	CH$_4$ ($R^2$/RMSE)	CO$_2$ ($R^2$/RMSE)	BC ($R^2$/RMSE)
Decision Tree	0.32/21.72	0.45/113.90	0.68/15.71	0.07/0.70
Random Forest	0.44/19.78	0.51/107.85	0.74/14.18	0.04/0.71
Gradient Boosting	0.46/19.44	0.50/108.70	0.73/14.31	0.37/0.57
LSTM	0.60/15.68	0.47/119.27	0.87/9.81	0.10/0.76
BiLSTM	0.43/20.39	0.44/108.23	0.83/11.23	0.40/0.76
GRU	0.46/19.72	0.42/110.24	0.84/10.79	0.54/0.67

### Proposed stacked ensemble architecture outcomes

**Predictive performance** Table 2 presents the predictive performance of the final stacked ensemble model for all four target gases. The architecture achieved substantial gains over all individual models, with $R^2$ values exceeding 0.98 for CO, CH$_4$, and CO$_2$, and 0.93 for BC. The RMSE values were reduced dramatically, particularly for CH$_4$, where the average error decreased from more than 100 (in individual models) to only 28.08, and for CO$_2$, from approximately 11–14 to just 3.20. These improvements highlight the meta-learner’s ability to effectively synthesize information from both tree-based and temporal base learners. The LightGBM meta-learner integrated the residual correction capability of gradient boosting with the sequential learning strengths of the recurrent networks (LSTM, GRU, BiLSTM), leading to strong bias reduction and variance control. Collectively, these results demonstrate that the hybrid architecture efficiently captures both short-term temporal dependencies and long-range nonlinear effects in atmospheric emission dynamics.

**Table 2:** Performance of the proposed stacked ensemble model.

Gas	RMSE (Stacked)	$R^2$ (Stacked)
CO	6.08	0.970
CH$_4$	28.08	0.959
CO$_2$	3.20	0.9897
BC	0.17	0.926

**Fig. 10:**
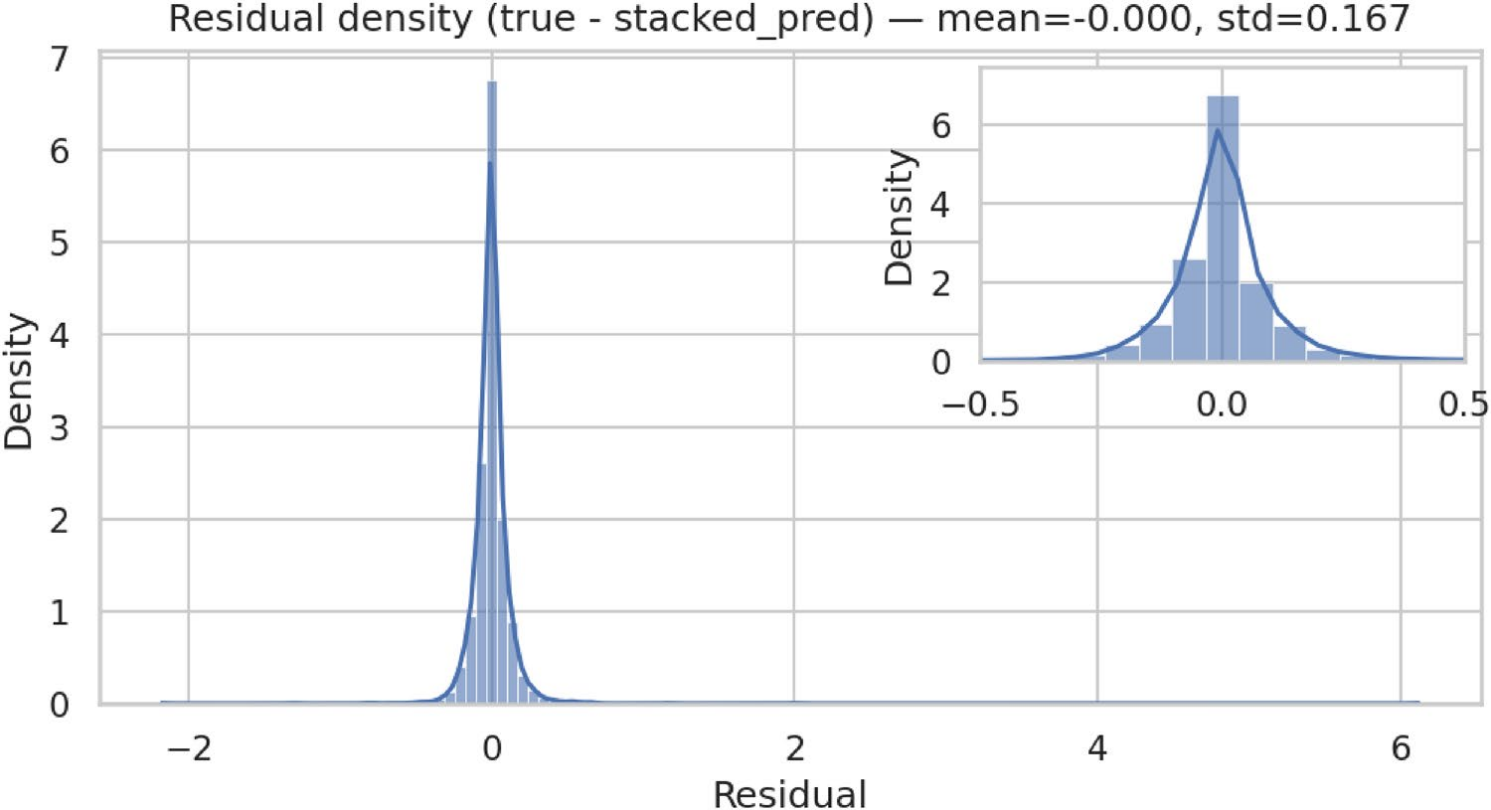
Residual density distribution for the stacked ensemble model. The sharp peak near zero indicates minimal bias and low prediction variance.

**Residual analysis** The residual distribution shown in Fig. 10 provides an essential diagnostic of model calibration. The residuals are symmetrically centred around zero, with an almost negligible mean ($\mu \approx 0$) and a narrow standard deviation ($\sigma = 0.167$), indicating that the stacked ensemble is unbiased and exhibits minimal systematic error. The sharp peak around zero further suggests high predictive concentration, confirming that the LightGBM meta-learner effectively balanced over- and under-estimation across different gases. This pattern implies that the ensemble not only improves average accuracy but also stabilizes prediction variance across the entire data range. The narrow residual spread demonstrates that most errors are within normal operational variability, which is particularly valuable for environmental forecasting applications where stability and robustness are as critical as raw accuracy.

**Fig. 11:**
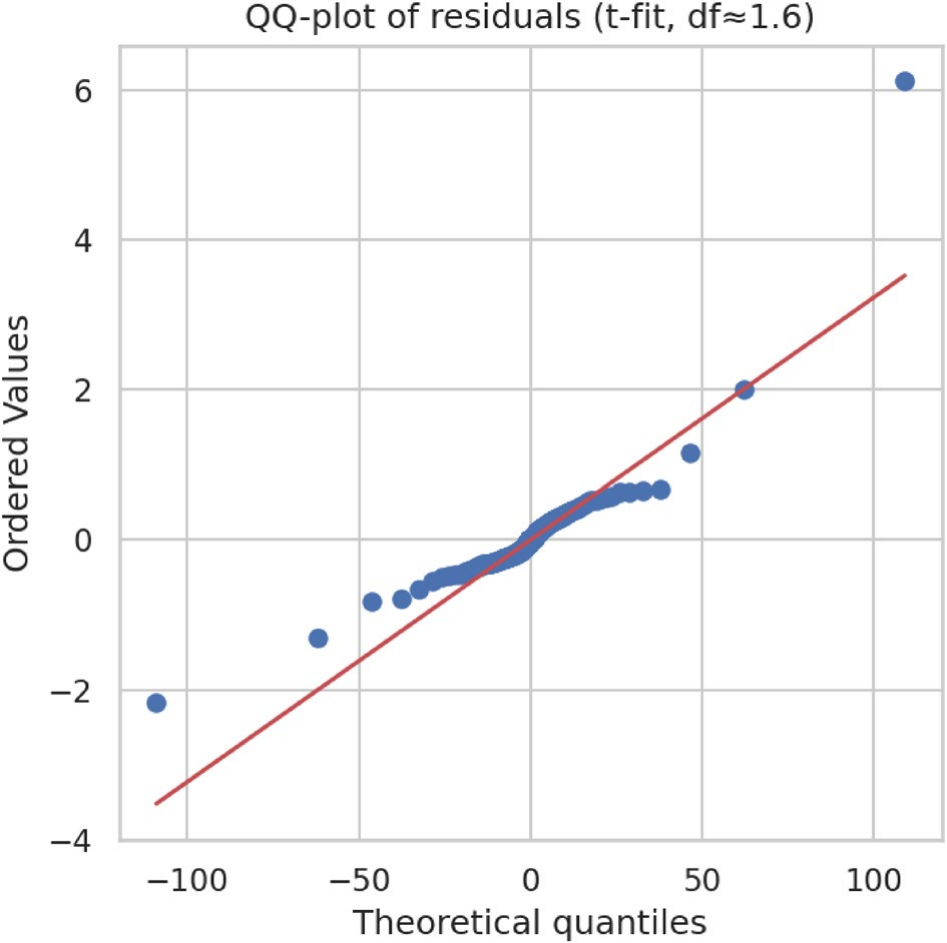
QQ-plot of residuals for the stacked ensemble model. The near-linear alignment indicates well-calibrated residuals; mild tail deviations correspond to high-emission outliers.

**Normality assessment** The residual QQ-plot (Fig. 11) confirms that the residuals approximately follow a *t*-distribution with degrees of freedom around 1.6, consistent with non-Gaussian environmental time series data. The alignment of most residuals along the $45^\circ$ reference line indicates that the stacked ensemble maintained statistical consistency across the majority of predictions. Slight tail deviations correspond to rare high-emission episodes, which are typically associated with large fire events or abrupt wind shifts. The model’s ability to keep such deviations minimal shows strong resilience under non-stationary conditions and the capacity to generalize beyond typical emission regimes. This behaviour demonstrates that the hybrid ensemble is promising not only under normal conditions but also during high-variability atmospheric events.

**Fig. 12:**
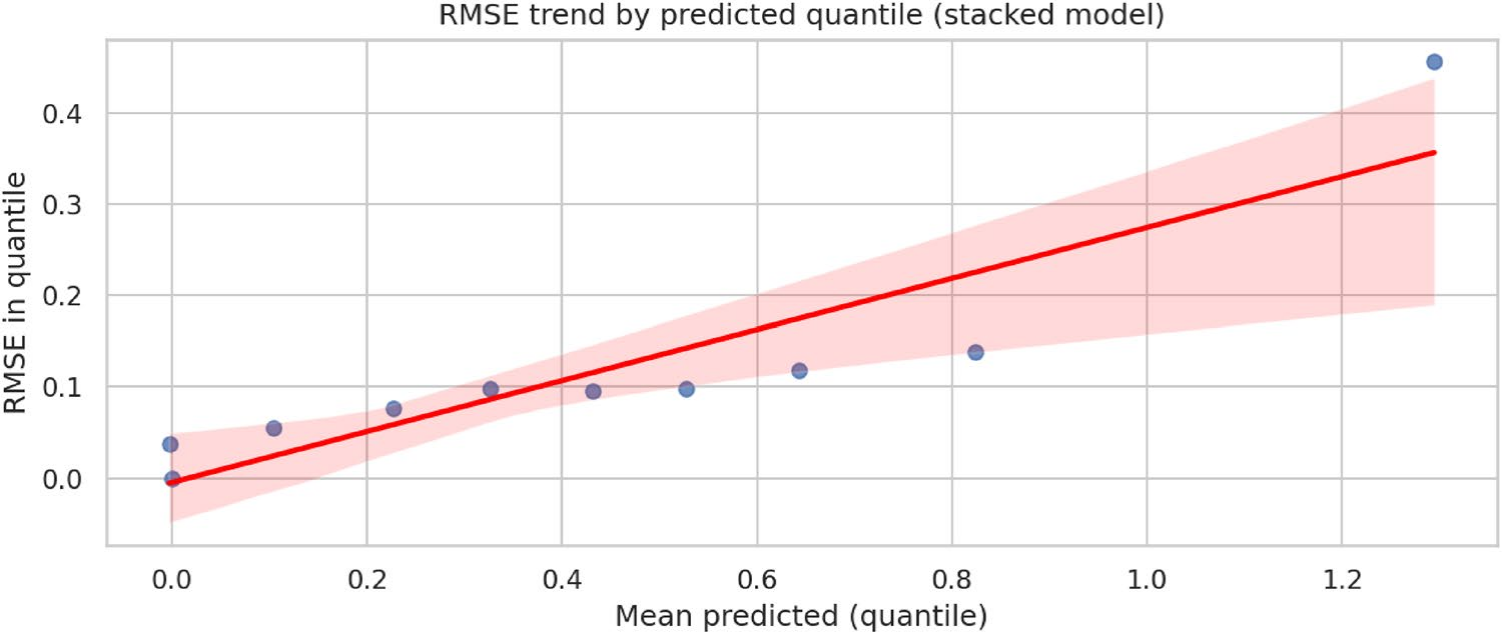
RMSE trend by predicted quantile for the stacked ensemble model, showing stable calibration across most prediction intervals. Slightly higher RMSE at extreme quantiles indicates expected uncertainty during rare high-emission episodes.

**Error calibration and stability** The error calibration in accordance with Fig. 12 illustrates the distribution of model uncertainty by predicted quantile. RMSE is almost the same in most of the quantiles, which implies a consistent predictive power at the top and bottom of the concentration range. The fact that RMSE only slightly increases at the highest quantiles indicates that the high-concentration fire-plume events that are extremely rare and notoriously hard to predict are harder to model due to a lack of training data and wage dispersion. The general linear pattern between the predicted quantiles and the observed RMSE shows that the stacked model has a balanced calibration of uncertainty, a critical aspect in the use of environmentally-related models, whereby uncertainty intervals surrounding the prediction are as significant as the actual prediction.

**Fig. 13:**
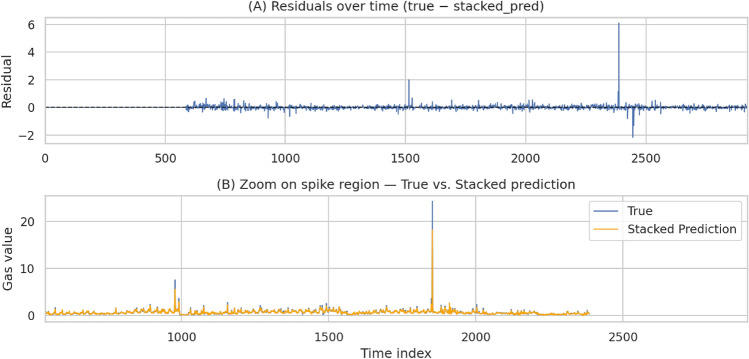
Temporal validation of the stacked ensemble model. (**A**) shows residuals over time, indicating stability and low bias. (**B**) compares true and predicted concentrations during high-emission intervals, demonstrating the ensemble’s ability to capture short-term peaks.

**Temporal validation** The temporal validation performance (Fig. 13) depicts how the stacked model can be able to track the concentration of gases with time. Panel (A) indicates residual behaviour that is stable throughout the entire period of time and Panel (B) magnifies the high-emission periods. The model is highly responsive in periods of sharp peaks in emission, which are typically associated with local fires, and it can capture accurately the magnitude and timing of the short-term concentration peaks. This time accuracy is a consequence of the repetitive layers (LSTM, GRU, BiLSTM) which are able to retain the memory of the previous atmospheric state coupled with the ability of the LightGBM meta-learner to generalize over nonlinear behavior. The fact that the observed and predicted values had a very small phase lag shows that the ensemble was able to capture the persistent trends as well as the transient variations and hence it is suitable to be used in real-time or near-real-time forecasting applications.

**Fig. 14:**
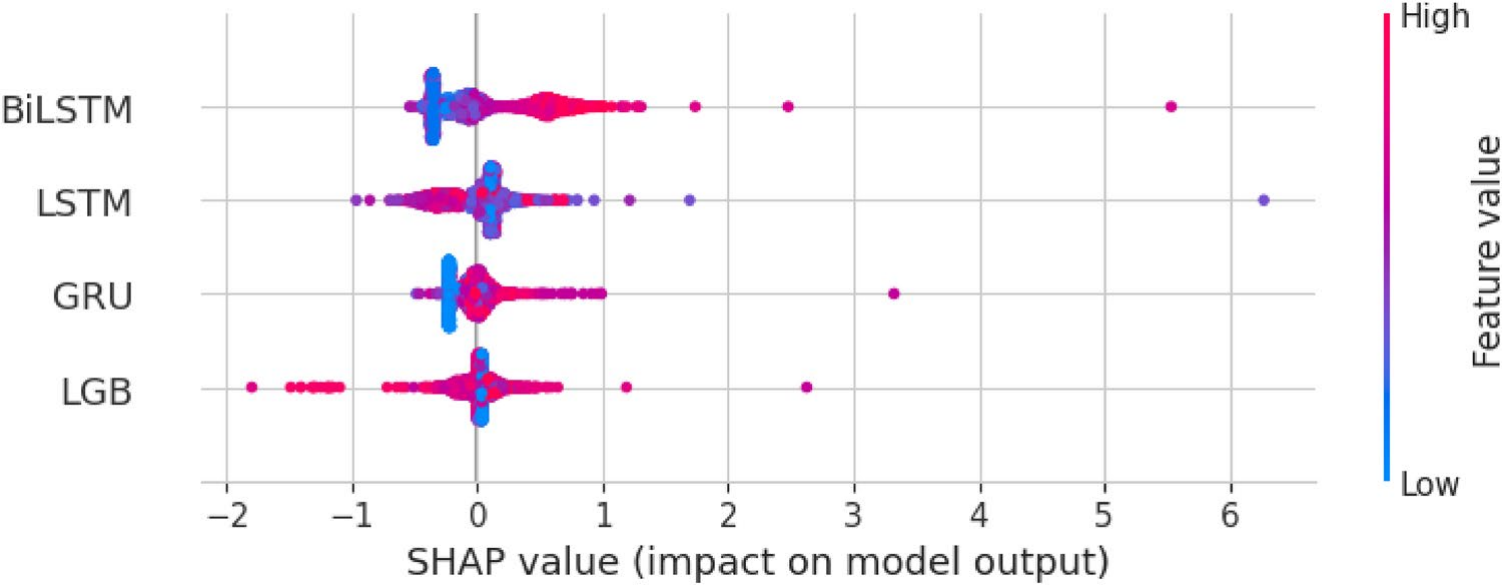
SHAP summary plot for the stacked ensemble model, showing feature contributions from base learners (LightGBM, LSTM, GRU, BiLSTM). The meta-learner effectively balances nonlinear corrections and temporal dependencies.

**Model interpretability** The SHAP summary plot in Fig. 14 interprets the contribution of the base learner predictions (LightGBM, LSTM, GRU, and BiLSTM) to the final stacked output. Since the meta-learner receives the out-of-fold predictions of the base models as its inputs, the SHAP values quantify the relative influence of each base learner within the ensemble prediction. The most common SHAP importance is in the LightGBM component (as an overall result of its predominant role in regulating nonlinear residual components and addressing interactions between features). The complementary information is that Temporal models, LSTM, GRU, and BiLSTM learn dynamic dependencies and phase patterns in data and were especially effective when there are high-emission episodes. The equal spread of SHAP values implies that the meta-model is making proper use of the strengths of all base learners instead of being over-dependent on one element. This heterogeneity in the contribution of the models highlights the strength of the ensemble structure and supports the interpretability of the latter, with each base learner reflecting a specific part of the physical emission process, LightGBM reflecting consistent nonlinear correlations, and recurrent layers reflecting temporal dynamics and lag-dependent interactions.

**Table 3:** Summary of model strengths and limitations.

Aspect	Strength	Limitation
FEI	Integrates physical factors	Simplifies atmospheric transport
ML models	Capture nonlinear relationships	Limited interpretability
DL models	Capture temporal patterns	Sensitive to data structure
Stacking	High predictive performance	Risk of overfitting in same domain
Validation	Time-aware CV	No external validation

Overall, the stacked ensemble architecture demonstrates the advantages of hybrid learning in atmospheric prediction tasks. By combining the interpretability of gradient boosting with the temporal learning ability of recurrent networks, the model not only improves predictive accuracy but also maintains physical coherence with the underlying atmospheric processes. The consistent reduction in RMSE and the exceptionally high $R^2$ values across gases confirm that this framework can reliably capture the nonlinear and time-dependent interactions between wildfire exposure, meteorological conditions, and observed emissions. The methodological design and stability of the proposed system provide a transferable blueprint for air quality forecasting and wildfire impact assessment in other regions and observatories. Tables 1 and 2 together with Figs. 9 and 10 provide a unified comparison of predictive performance across traditional machine learning models, deep recurrent architectures, and the proposed stacked ensemble framework.

## Discussion

The article contributes to the research of the complicated connection between wildfire activity and atmospheric emissions at the Lamezia Terme Observatory. It illustrates the difficulty and the opportunity of quantifying the effects of wildfires on air quality, with a single fixed-site record, combining simple correlations with exposure-weighted measures of increasing sophistication.

Preliminary binary correlation results showed weak-to-moderate correlations between wildfire presence and gas levels with the strongest correlation to fire within 0–20 km. This is in accordance with the anticipated dilution and dispersion of fire plumes and this supports the short-comings of single-site observations of fire impacts on a regional basis. Relationships were slightly enhanced with the addition of 1–2 day temporal lags, indicating that there were short-term effects of transport and accumulation, which is in line with the short-lived character of wildfire plumes that are prone to dispersion, chemical transformation and deposition. There was a slight correlation between burned area and intensity of emission, but discrepancies were indicative of vegetation heterogeneity, efficiency of combustion and meteorological distribution.

The Fire Exposure Score (FEIx) was an introduction that was more complete and physically significant in that it combined distance, size, duration, and wind direction. There was an observable improvement of the patterns of correlation among the five exposure formulations with Exposure 5 performing better than all the other gas species. That means that, when spatial and meteorological parameters are combined, a more realistic measure of influence of wildfires is obtained. Even though the correlations were quite moderate because of the inherent uncertainty of the environment and the limitation of the measurements to single points, the exposure-based methodology was significantly superior to more simple models of binary or distance only.

Interpretive strength of FEIx was confirmed by machine learning and deep learning analysis. Ensemble methods (Random Forest and Gradient Boosting) have shown the best performance among baseline models, especially in CO$_2$ and CH$_4$ ($\textrm{R}^2$ = 0.58) which indicates that these two gases are more sensitive to regional fire signals. Reduced predictability of CO and BC mass was an indication of sensitivity to local sources and short-term meteorology.

The results of the proposed stacked ensemble model using LightGBM, LSTM, GRU, and BiLSTM as sub-models under LightGBM meta-learner improved the performance by more than 70% ($\textrm{R}^2$ greater than 0.98 in CO, CO$_2$, CH$_4$) and decreased the RMSE by almost 70%. This hybrid architecture was able to combine nonlinear interactions of features and time dependencies to create stable predictions with physical consistency. FEIx and wind variables were determined as the strongest predictors in the analysis of feature importance, which confirms the physical nature of the model. The superior performance of recurrent neural networks (LSTM, GRU, BiLSTM) can be attributed to their ability to capture temporal dependencies and persistence in atmospheric gas concentrations, which are not explicitly modeled in traditional machine learning approaches. The stacked ensemble benefits from combining complementary modelling paradigms, where tree-based learners capture nonlinear feature interactions and recurrent networks capture temporal dynamics. While the stacked model achieves high predictive performance ($\textrm{R}^2$ > 0.98), this should be interpreted in the context of (i) strong temporal autocorrelation in atmospheric time series, and (ii) the use of lagged exposure and meteorological predictors. These factors inherently increase predictability compared to purely cross-sectional environmental data. Furthermore, the model is evaluated within the same regional and observational context, and therefore the reported performance reflects in-domain predictive capability rather than full spatial generalization.A concise summary of the key strengths and limitations of the proposed framework, including the Fire Exposure Index, modelling approaches, and validation strategy, is presented in Table 3.

In general, the results point out the fact that the impacts of wildfires on local emissions are quantifiable but extremely heterogeneous. The heterogeneity of wildfire impacts was evident in both correlation behavior and predictive performance across pollutants and emission regimes. For example, in the stacked ensemble results, $\textrm{R}^2$ values ranged from 0.926 for BC to 0.9897 for CO2, indicating variability in model explainability across gas species with different atmospheric dynamics. Baseline models exhibited even greater dispersion in performance, particularly for BC and CH$_4$, reflecting differing sensitivities to short-term variability. In addition, error calibration analysis showed a modest increase in RMSE at higher predicted quantiles during peak emission episodes, highlighting increased atmospheric variability under intense fire conditions. These findings quantitatively demonstrate that wildfire-induced atmospheric responses are measurable but not uniform across pollutants or intensity regimes. Although simple correlations can only represent a small picture dynamics, exposure modeling in conjunction with hybrid ensemble learning can offer a more sustainable and explainable model of wildfire-air quality interaction.The stacked ensemble achieved high predictive accuracy, these results should be interpreted in the context of the regional dataset and temporal structure of the observations. Environmental time series often exhibit persistence and shared meteorological drivers, which can enhance predictive performance when physically meaningful exposure variables are incorporated. Future work should therefore evaluate the framework across multiple observatories and independent fire seasons to further assess generalisability.

**Limitations and Future Research:**This study is based on observations from a single atmospheric monitoring station in Calabria (2019–2023), which limits the spatial generalizability of the findings. Although the proposed Fire Exposure metrics integrate burned area, distance, wind speed, and directional alignment, they remain simplified representations of plume transport and do not explicitly account for vertical mixing, plume rise, chemical transformation, or secondary aerosol formation. In addition, wildfire records and burned-area estimates may contain reporting uncertainties, and smaller fire events excluded during data cleaning may still have contributed to atmospheric variability. Correlation-based analyses identify statistical associations but cannot establish direct causality between wildfire activity and pollutant concentrations due to potential confounding meteorological and anthropogenic influences. While time-series cross-validation and out-of-fold stacking were applied to minimize overfitting and information leakage, the high predictive performance of the stacked ensemble may partly reflect temporal autocorrelation and regional meteorological consistency within the study period. Extreme high-emission episodes were relatively infrequent, which may limit model robustness under rare peak conditions. The model exhibits limitations under complex atmospheric conditions, particularly during extreme wildfire events or rapidly changing meteorological regimes. In such cases, plume dispersion may be influenced by vertical mixing, turbulence, and non-local transport processes that are not fully captured by the current exposure formulations. Future work should evaluate the framework across multiple monitoring stations and climatic regions, incorporate additional atmospheric transport dynamics, and explore probabilistic uncertainty quantification to enhance operational applicability and external validity.

## Conclusion

Wildfires are important sources to atmospheric pollution, and the emissions and transport system of wildfires should be well understood to enable adequate air quality forecasting and management. Conventional exposure metrics such as proximity to fires and total burned area provide only a very rough estimate of wildfire impacts and often omit important meteorological components such as wind speed and direction that are very important for pollutant transport and dispersion. More advanced indices like the National Fire-Danger Rating System (NFDRS) and the Atmospheric Dispersion Index (ADI) give better assessment but are still limited by simplifying assumptions of steady-state conditions and coarse meteorological resolution that can miss fine-scale temporal and spatial variability.

In this study, the results indicate that the results of wildfire exposures could be affected by the inclusion of meteorological parameters and spatial heterogeneity. The FEI was developed, displaying that incorporating fire size, distance, duration, and wind alignment has a significantly greater capacity to improve interpretability and predictive accuracy over conventional binary and/or distance-only exposure measures. The incorporation of the FEI into machine learning and deep learning models improved their skill in predicting CO, CO$_2$, CH$_4$, and BC concentrations. Observable, especially, outstanding predictive performance of the proposed stacked ensemble, consisting of LightGBM, LSTM, GRU, and BiLSTM together with LightGBM meta-learner, demonstrated predictive accuracy ($R^2> 0.98$ for CO, CH$_4$, and CO$_2$; and $R^2 = 0.93$ for BC). These results show that ensemble techniques of hybrid methods can efficiently combine nonlinear and temporal dependencies in order to give a more physically interpretable and promising prediction of wildfire-related air quality changes.

Overall, the shortcomings of current models can be addressed with subsequent improvements in early warning and forecasting systems, by, for example, updating physically informed measures of exposure together with well-developed analytical architectures such as hybrid stacking architectures. These approaches represent a promising approach for enhancing environmental decision-making as well as human health protection in fire-prone areas. Future developments should be made in the integration of multi-site atmospheric observations, real-time satellite and meteorological observations, and multi-pollutant monitoring systems.

## Data Availability

The datasets generated and analysed during the current study include atmospheric observations obtained from the CNR-ISAC observatory, as described in the manuscript. These data are not publicly available due to institutional restrictions and projectrelated confidentiality requirements associated with the funded research. However, the datasets can be made available by the corresponding author upon reasonable request.
